# Genetic diversity and prevalence of emerging Rickettsiales in Yunnan Province: a large-scale study

**DOI:** 10.1186/s40249-024-01213-4

**Published:** 2024-07-10

**Authors:** Chun-Hong Du, Rong Xiang, Shuang-Shuang Bie, Xing Yang, Ji-Hu Yang, Ming-Guo Yao, Yun Zhang, Zhi-Hai He, Zong-Ti Shao, Chun-Feng Luo, En-Nian Pu, Yu-Qiong Li, Fan Wang, Zhi Luo, Chao-Bo Du, Jie Zhao, Miao Li, Wu-Chun Cao, Yi Sun, Jia-Fu Jiang

**Affiliations:** 1 Yunnan Key Laboratory for Zoonosis Control and Prevention, Yunnan Institute for Endemic Diseases Control and Prevention, Dali, 671000 PR China; 2 State Key Laboratory of Pathogen and Biosecurity, Academy of Military Medical Sciences, Beijing, 100071 PR China; 3 Department of Medical Microbiology and Immunology, School of Basic Medicine, Dali University, Dali, 671000 PR China

**Keywords:** Rickettsiales, *Candidatus* Rickettsia longicornii, Tick, Small mammal, China

## Abstract

**Background:**

Rickettsia and related diseases have been identified as significant global public health threats. This study involved comprehensive field and systematic investigations of various rickettsial organisms in Yunnan Province.

**Methods:**

Between May 18, 2011 and November 23, 2020, field investigations were conducted across 42 counties in Yunnan Province, China, encompassing small mammals, livestock, and ticks. Preliminary screenings for Rickettsiales involved amplifying the 16S rRNA genes, along with additional genus- or species-specific genes, which were subsequently confirmed through sequencing results. Sequence comparisons were carried out using the Basic Local Alignment Search Tool (BLAST). Phylogenetic relationships were analyzed using the default parameters in the Molecular Evolutionary Genetics Analysis (MEGA) program. The chi-squared test was used to assess the diversities and component ratios of rickettsial agents across various parameters.

**Results:**

A total of 7964 samples were collected from small mammals, livestock, and ticks through Yunnan Province and submitted for screening for rickettsial organisms. Sixteen rickettsial species from the genera *Rickettsia*, *Anaplasma*, *Ehrlichia*, *Neoehrlichia*, and *Wolbachia* were detected, with an overall prevalence of 14.72%. Among these, 11 species were identified as pathogens or potential pathogens to humans and livestock. Specifically, 10 rickettsial organisms were widely found in 42.11% (24 out of 57) of small mammal species. High prevalence was observed in *Dremomys* samples at 5.60%, in samples from regions with latitudes above 4000 m or alpine meadows, and in those obtained from Yuanmou County. *Anaplasma phagocytophilum* and *Candidatus* Neoehrlichia mikurensis were broadly infecting multiple genera of animal hosts. In contrast, the small mammal genera *Neodon*, *Dremomys*, *Ochotona*, *Anourosorex*, and *Mus* were carrying individually specific rickettsial agents, indicating host tropism. There were 13 rickettsial species detected in 57.14% (8 out of 14) of tick species, with the highest prevalence (37.07%) observed in the genus *Rhipicephalus*. Eight rickettsial species were identified in 2375 livestock samples. Notably, six new Rickettsiales variants/strains were discovered, and *Candidatus* Rickettsia longicornii was unambiguously identified.

**Conclusions:**

This large-scale survey provided further insight into the high genetic diversity and overall prevalence of emerging Rickettsiales within endemic hotspots in Yunnan Province. The potential threats posed by these emerging tick-borne Rickettsiales to public health warrant attention, underscoring the need for effective strategies to guide the prevention and control of emerging zoonotic diseases in China.

**Graphical Abstract:**

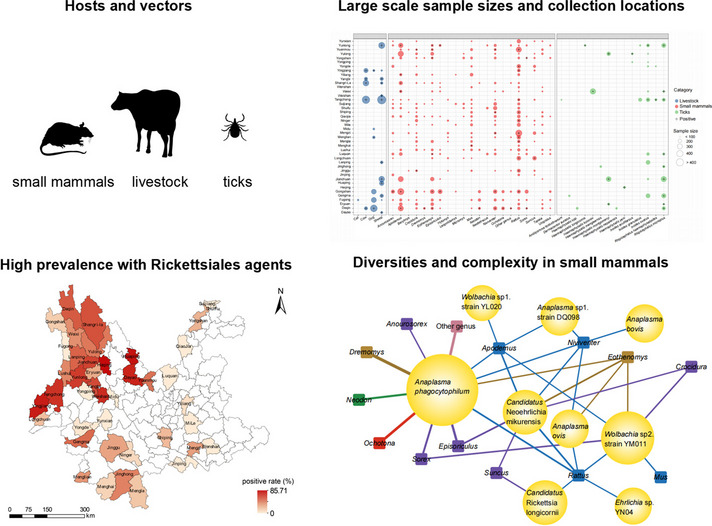

**Supplementary Information:**

The online version contains supplementary material available at 10.1186/s40249-024-01213-4.

## Background

The order Rickettsiales comprises a major group of obligate intracellular Gram-negative bacteria responsible for various human diseases [1]. This order primarily includes *Rickettsia*, *Anaplasma*, *Ehrlichia, Neoehrlichia, Wolbachia,* and *Orientia* genera, each of which preferentially infects different vectorial invertebrate arthropods like ticks, fleas, mosquitoes, and mites [2]. Rickettsiales are highly prevalent and globally distributed across various arthropod vectors, animals, and human [3, 4]. Tick-associated rickettsioses are recognized as significant emerging zoonoses worldwide. Pathogens from the genera *Anaplasma, Ehrlichia,* and *Rickettsia* have been documented in parasitic ticks collected from humans [5].

Currently, there are at least 25 known tick species capable of transmitting pathogenic *Rickettsia* species [6]. Eight formally described species within the genus *Anaplasma* [7, 8] and 4 species within the genus *Ehrlichia* that are extensively distributed among terrestrial wild animal hosts [4, 9, 10]. In China, at least 11 species of tick-associated Rickettsiales known to cause human disease have been confirmed [11–14]. Clinical manifestations of tick-associated rickettsial diseases range from mild symptoms like fever, malaise, headache, myalgia, lymphadenopathy, rash, scabs, and gastrointestinal symptoms to severe complications such as renal or respiratory failure, respiratory acidosis, hyponatremia, pericarditis, septic shock, pneumonia, pleural effusion, hemorrhage, neurological complications, and potentially death [11–14].

Over the past three decades, advances in molecular diagnostic techniques have led to the rapid and precise identification of emerging pathogens among tick-associated rickettsial agents, as well as some *Candidatus* species of rickettsial agents worldwide [14–19]. In particular, novel rickettsioses and newly recognized natural foci often emerge unexpectedly. The rising popularity of ecotourism, extensive global travel, and the frequent migration of wild animal populations along with parasitic ticks have significantly contributed to the increasing incidence of tick-associated rickettsioses. Therefore, addressing this issue and reducing the burden of human rickettsioses presents significant challenges for public health authorities, physicians, veterinarians, and scientists.

In China, numerous reports have documented the distribution and co-circulation of Rickettsiales bacteria among ticks, wild animals, livestock, and febrile patients [20–22]. Yunnan Province, located in Southwest China and bordering the Indo-Chinese peninsula, is characterized by diverse eco-climatic zones and is recognized as a biodiversity hotspot. This region is of unique medical significance for assessing potential spillover vector-borne zoonoses and the emergence of new pathogens. Previous surveys have highlighted the high diversity of Rickettsiales in limited tick and small mammals samples collected from Yunnan [20, 23–26]. It is speculated that the host and vector species of tick-associated Rickettsiales in Yunnan Province are abundant and widely dispersed. In this study, extensive field and systematic investigations of multiple rickettsial species were conducted using molecular detection methods to reveal the true nature of Rickettsiales infections and their potential threats to public health.

## Methods

### Study sites and sample collection

Between May 18, 2011 and November 23, 2020, field investigations covering small wildlife mammals, domestic animal hosts, and tick vectors were conducted across 42 counties of Yunnan Province, encompassing various geographical terrains such as alpine meadows, cultivated lands, bushes, woodlands, coniferous forests, broadleaved forests, mixed forests, bamboo forests, residential areas, and dry-hot valleys, spanning an altitude gradient ranging from 383 to 4000 m above sea level.

Small mammals were sampled using the baited snap night-trap method. Morphological identification of captured animals was performed by experienced biologists and subsequently confirmed through sequence analysis of partial cytochrome oxidase I (*COI*) gene of suspected rodent species, followed by aseptic dissection to obtain liver and spleen tissue samples [27]. Livestock were sampled for whole blood collection via jugular vein puncture using EDTA anticoagulant tubes. Domestic mammal-associated ticks were collected by manual scratching, while host-seeking ticks were obtained through flag-sweeping on vegetation at the same sampling sites. Morphological identification of ticks was conducted by trained entomologists and subsequently validated using molecular evidence from *COI* genes [28]. All collected samples, including blood, tissues, and ticks, were transported to the laboratory under cold-chain conditions and stored at -80 °C until further processed.

### DNA extraction, PCR and sequencing

DNA extraction from host blood samples and small mammal tissue samples was conducted based on the instructions provided by the manufacturer with the TIANamp Genomic DNA Kit (TIANGEN, Cat. No. 4992199). The identified ticks underwent surface sterilization with 70% alcohol, followed by drying and resin coating in PBS before being crushed. Buffer ATL (a tissue lysis buffer) and protease K were added to the homogenized liquid to facilitate tissue lysis. Subsequently, after centrifugation in a water bath, buffer ATL and anhydrous ethanol were added for DNA extraction. The DNA from individual ticks was extracted using the DNeasy Blood & Tissue Kit (Qiagen, Cat. No. 69504), and the extracted DNA was stored in a freezer set at -80 °C for future use.

For screening the presence of bacterial DNA, including *Rickettsia* spp., *Anaplasma* spp*.*, *Ehrlichia* spp*.*, *Neoehrlichia* spp*.*, and *Wolbachia* spp., primers targeting 16S rRNA of *Rickettsiaceae* and *Anaplasmataceae* agents were used. The primers used were Eh-out1 (5’-TTGAGAGTTTGATCCTGGCTCAGAACG-3’), 3-17pan (5’-TAAGGTGGTAATCCAGC-3’), and Eh-out2 (5’-CACCTCTACACTAGGAATTCCGCTATC-3’) [29]. Positive controls comprised of DNA templates from several previously confirmed positive samples. All positive PCR samples underwent purification using the Agarose Gel DNA Purification Kit (TaKaRa, Dalian, China) and were directly forwarded to Kunming Sangon Biological Engineering Technology and Services Co., Ltd. (Kunming, China) for sequencing.

The sequences were successfully assembled using the CLC Genomics Workbench (version 3.6.11, www.clcbio.com). Based on preliminary sequencing results, positive samples carrying human pathogenicity-associated pathogens or potential novel variants underwent further confirmation using nested PCR assays targeting related functional protein genes with previously reported primers (Table S1) [30–36].

### Sequence comparisons and phylogenetic analyses

Sequence comparisons were conducted using the Basic Local Alignment Search Tool (BLAST) available on the NCBI website (https://blast.ncbi.nlm.nih.gov/Blast.cgi) to analyze species and gene types. All sequences were aligned using Clustal W with default parameters in the Molecular Evolutionary Genetics Analysis (MEGA) program (version 6.0, The Pennsylvania State University, PA, USA). Phylogenetic relationships were inferred using the neighbor-joining method based on 1000 bootstrapped datasets. The statistical support for individual nodes was indicated by posterior probability values.

### Statistical analyses

The chi-squared test or Fisher’s exact method was used to analyze the diversities and component ratios of rickettsial agents across various parameters, their distribution among different host species, habitats, and other potential influencing factors. Variables with a *P* value of < 0.05 were deemed statistically significant. All analyses were performed using SPSS (version 17.0, SPSS Inc., Chicago, IL).

## Results

### Diverse small mammals, livestock and ticks involved in *Rickettsiales* natural cycles

**Fig. 1:**
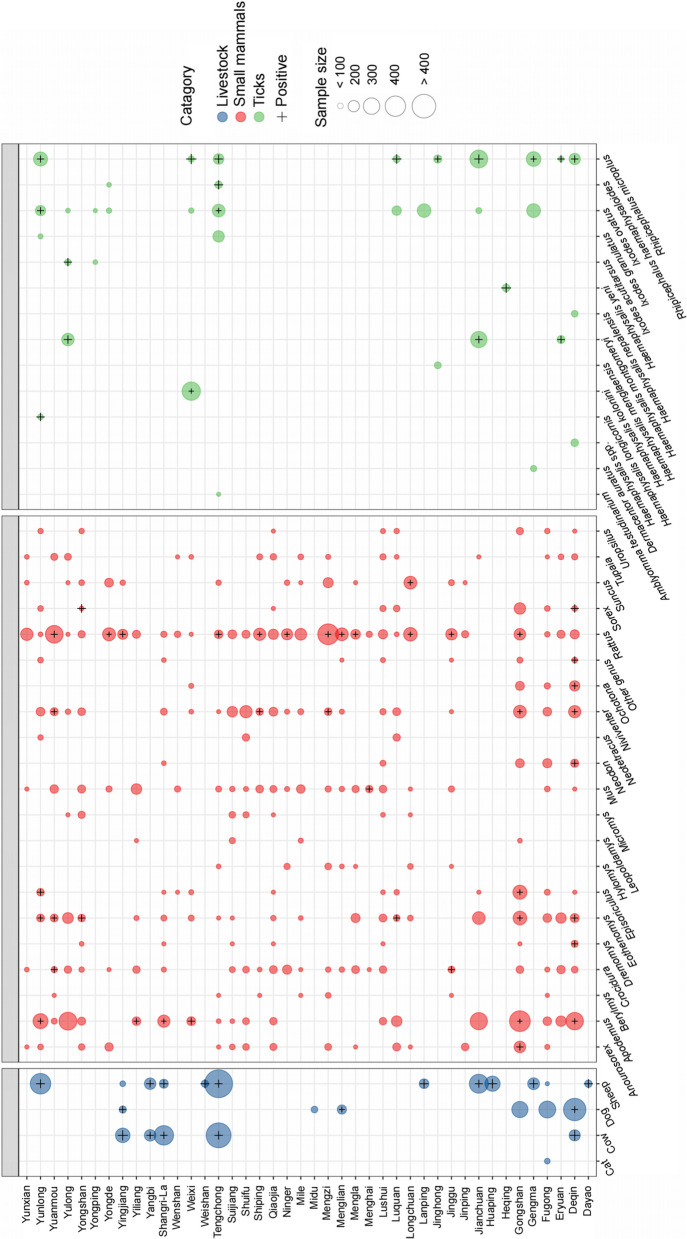
Distribution of different species of samples and positive samples in different counties in Yunnan Province. Blue, red and green circles represent samples of livestock, small mammals and ticks, “ + ” represent sites, hosts and vectors with positive results for rickettsiae. The circles size of symbols represents the number of samples

Between May 18, 2011 and November 23, 2020, a total of 7964 organisms were collected from 42 investigation counties (Table 1). Among them, 4330 small mammals were identified, representing 57 species across 21 genera, 10 families, and 4 orders. *Rattus tanezumi* emerged as the dominant species with the highest constituent ratio (720/4330, 16.63%) and exhibited the broadest distribution across 28 counties (Fig. 1, Table S2). Livestock samples comprised of 2375 individuals, representing 4 species, with goat (*Capra aegagrus*) as the predominant species (446/2375, 18.78%). Of the 1259 ticks collected, 829 were parasitized ticks and 430 were host-seeking ones, classified into 14 species under 5 genera of Ixodidae. *Rhipicephalus microplus* ranked as the dominant species (457/1259, 36.30%), followed by *Ixodes ovatus* (302/1259, 23.99%) (Fig. 1, Table S2).

**Table 1:** Overall prevalence of animals and vectors from different counties with emerging rickettsiae in Yunnan Province

Sampling counties	No. of positive/Tested (%)			
	Small mammals	Livestock	Ticks	Overall sample
Tengchong	2/39 (5.13)	276/758 (36.41)	30/166 (18.07)	308/963 (31.98)
Fugong	0/130 (0.00)	0/139 (0.00)	—	0/269 (0.00)
Shuifu	0/128 (0.00)	—	—	0/128 (0.00)
Qiaojia	0/126 (0.00)	—	—	0/126 (0.00)
Luquan	0/120 (0.00)	—	—	0/120 (0.00)
Suijiang	0/95 (0.00)	—	—	0/95 (0.00)
Mile	0/92 (0.00)	—	—	0/92 (0.00)
Yunxian	0/68 (0.00)	—	—	0/68 (0.00)
Jinping	0/27 (0.00)	—	—	0/27 (0.00)
Wenshan	0/17 (0.00)	—	—	0/17 (0.00)
Yunlong	8/177 (4.52)	110/212 (51.89)	24/127 (18.90)	142/516 (27.52)
Jianchuan	0/206 (0.00)	55/175 (31.43)	126/282 (44.68)	181/663 (27.30)
Deqin	22/346 (6.36)	75/304 (24.67)	10/70 (14.29)	107/720 (14.86)
Gengma	—	25/54 (46.30)	6/180 (3.33)	31/234 (13.52)
Yingjiang	3/38 (7.89)	89/109 (81.65)	—	92/147 (62.59)
Lanping	—	15/30 (50.00)	0/77 (0.00)	15/107 (14.02)
Shangri-La	1/91 (1.10)	47/211 (22.27)	—	48/302 (15.89)
Weixi	4/40 (10.00)	—	9/174 (5.17)	13/214 (6.07)
Yulong	0/224 (0.00)	—	27/72 (37.50)	27/296 (9.12)
Lushui	1/114 (0.88)	—	9/40 (22.50)	10/154 (6.49)
Mengla	1/80 (1.25)	1/26 (3.85)	—	2/106 (1.89)
Eryuan	0/115 (0.00)	—	5/20 (25.00)	5/135 (3.70)
Gongshan	12/680 (1.76)	0/120 (0.00)	—	12/800 (1.50)
Yongde	1/119 (0.84)	—	0/6 (0.00)	1/125 (0.80)
Yuanmou	25/226 (11.06)	—	—	25/226 (11.06)
Menglian	17/303 (5.61)	—	—	17/303 (5.61)
Mengzi	1/21 (4.76)	—	—	1/21 (4.76)
Jinggu	3/75 (4.00)	—	—	3/75 (4.00)
Longchuan	3/157 (1.91)	—	—	3/157 (1.91)
Shiping	2/96 (2.08)	—	—	2/96 (2.08)
Menghai	1/86 (1.16)	—	—	1/86 (1.16)
Ninger	1/88 (1.14)	—	—	1/88 (1.14)
Yiliang	1/94 (1.06)	—	—	1/94 (1.06)
Yongshan	3/112 (2.68)	—	—	3/112 (2.68)
Midu	—	0/9 (0.00)	—	0/9 (0.00)
Huaping	—	68/103 (66.02)	—	68/103 (66.02)
Dayao	—	8/13 (61.54)	—	8/13 (61.54)
Weishan	—	7/12 (58.33)	—	7/12 (58.33)
Yangbi	—	24/100 (24.00)	—	24/100 (24.00)
Yongping	—	—	0/3 (0.00)	0/3 (0.00)
Heqing	—	—	12/14 (85.71)	12/14 (85.71)
Jinghong	—	—	2/28 (7.14)	2/28 (7.14)
Total	112/4330 (2.59)	800/2375 (33.68)	260/1259 (20.65)	1172/7964 (14.72)

— Means no samples were collected

### Ten species under five genera emerging *Rickettsiales* in small mammals

**Fig. 2:**
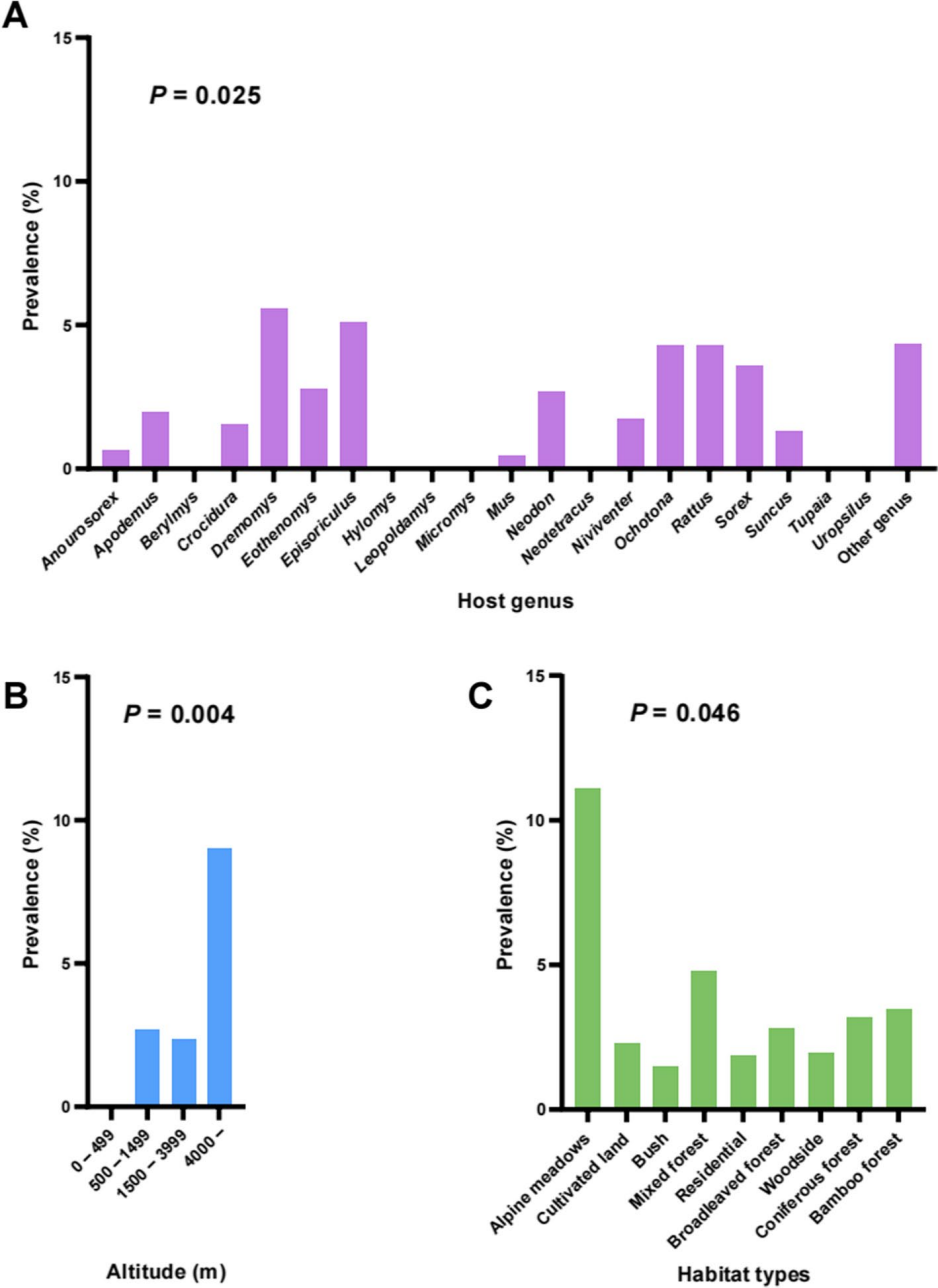
Comparison the positivity rate of rickettsiae in different altitudes, hosts, and habitats in Yunnan Province. **A** Host genus (*P* = 0.025) **B** Altitude (*P* = 0.004) **C** Habitat types (*P* = 0.046)

In total, 2.59% (112/4330) of sampled individuals and 24 out of 57 (42.11%) species of small mammals were infected with rickettsial agents, primarily concentrated in western counties of Yunnan Province. The highest prevalence of rickettsial agents was observed in Yuanmou County (11.06%), followed by Weixi County (10.00%), with significant differences (*P* < 0.001) (Table 1). Among the 21 identified genera, the genus *Rattus* exhibited the highest frequencies of rickettsial agent infections (*n* = 51), with *R. tanezumi* having a prevalence of 3.06%. The genus *Dremomys* had the highest prevalence (5.60%, 1/18). Significant differences in prevalence were observed among genera of small mammals (*P* = 0.025) (Fig. 2A). Furthermore, the highest prevalence of rickettsial agents was observed in areas with altitudes above 4000 m (*P* = 0.004) (Fig. 2B). Additionally, the prevalence of rickettsiae was higher in males than in females (*P* = 0.004). Small animals collected from alpine meadows exhibited the highest prevalence, followed by those in mixed forests (*P* = 0.046) (Fig. 2C).

**Fig. 3:**
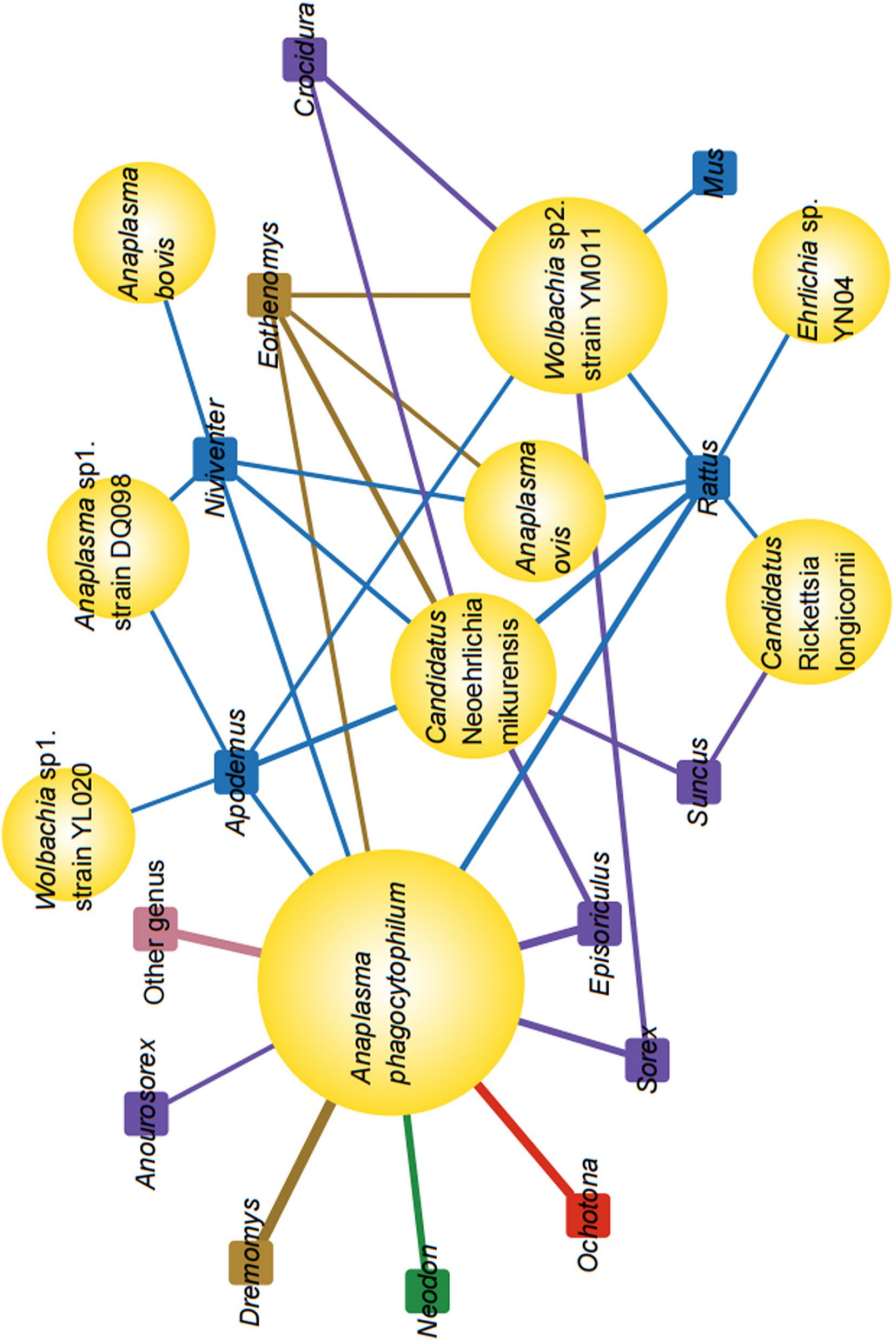
Host-rickettsial correlation network topology. Rectangles represent the genera of the small mammals; the size of the circle represents the level of rickettsial agents’ positivity and the thickness of the straight line represents the level of host infection with rickettsiae

To better understand the potential transmission of rickettsial agents in small mammals, we constructed host-rickettsial correlation networks. Among the small mammals studied, 21 genera were found in 10 families. Of these, 14 genera in 6 families of small animals were found to be harboring rickettsial agents. Some rickettsial agents were specifically found to infect only one genus, such as *Wolbachia* sp. strain YL020 in the genus *Apodemus, Anaplasma bovis* in the genus *Niniventer*, and *Ehrlichia* sp. YN04 in the genus *Rattus*. Conversely, the genera *Neodon, Dremomys, Ochatona,* and *Anourosorex* were found to carry *A. phagocytophilum*, and the genus *Mus* was detected to host only *Wolbachia* sp. strain YM011, indicating host tropism, a preference towards certain rickettsia. However, both *A. phagocytophilum* and *Candidatus* Neoehrlichia mikurensis (CNM) were detected in more than 7 genera of small animals (Fig. 3), indicating a broad host spectrum.

**Fig. 4:**
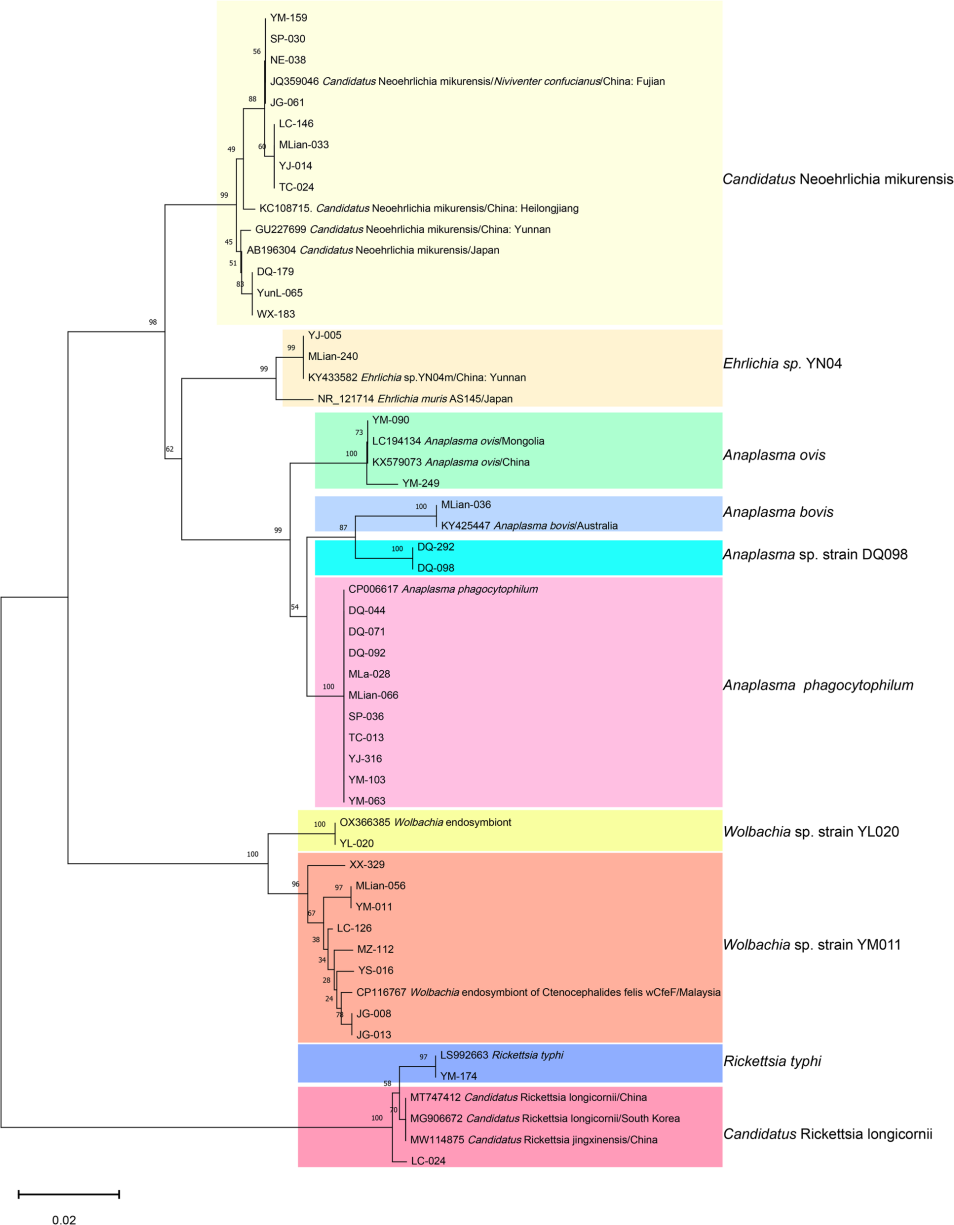
Phylogenetic tree based on partial sequences of 16S rRNA (660 bp) gene of the rickettsiae species derived from small mammals. Phylogenetic analysis was performed using the neighbor-joining method and trees were tested by bootstrapping (1000 replicates)

As revealed in Fig. 4, 10 different species of rickettsial agents were detected. Among these, *A. phagocytophilum, A. ovis,* CNM, and *Rickettsia typhi* were confirmed as human pathogens. CNM exhibited the highest positivity rate (1.04%, 45/4330), followed by *A. phagocytophilum* (0.97%, 42/4330) in small mammals. Notably, the sequences of samples DQ098 and DQ292 were identical and revealed the closest similarity (97.65%) to an uncultured *Anaplasma* sp. clone T7 (GenBank No. KU189193). This was provisionally termed *Anaplasma* sp. strain DQ098. Additionally, YL-020 and the other eight identical sequences represented by YM-011 had the highest similarities of 99.68% with OX366385 and 98.72% with CP116767, respectively, representing two distinct *Wolbachia* spp. (Table S3).

The constructed phylogenetic tree revealed that the rickettsial species detected in the small mammals clustered into five major groups: *Neoehrlichia, Ehrlichia, Anaplasma, Wolbachia*, and *Rickettsia* (Fig. 4). Within the *Wolbachia* cluster, the sequences identified were significantly different from known species and were categorized into two clades, termed *Wolbachia* sp. strain YL020 and *Wolbachia* sp. strain YM011.

### High prevalence of eight species emerging *Rickettsiales* in livestocks

**Fig. 5:**
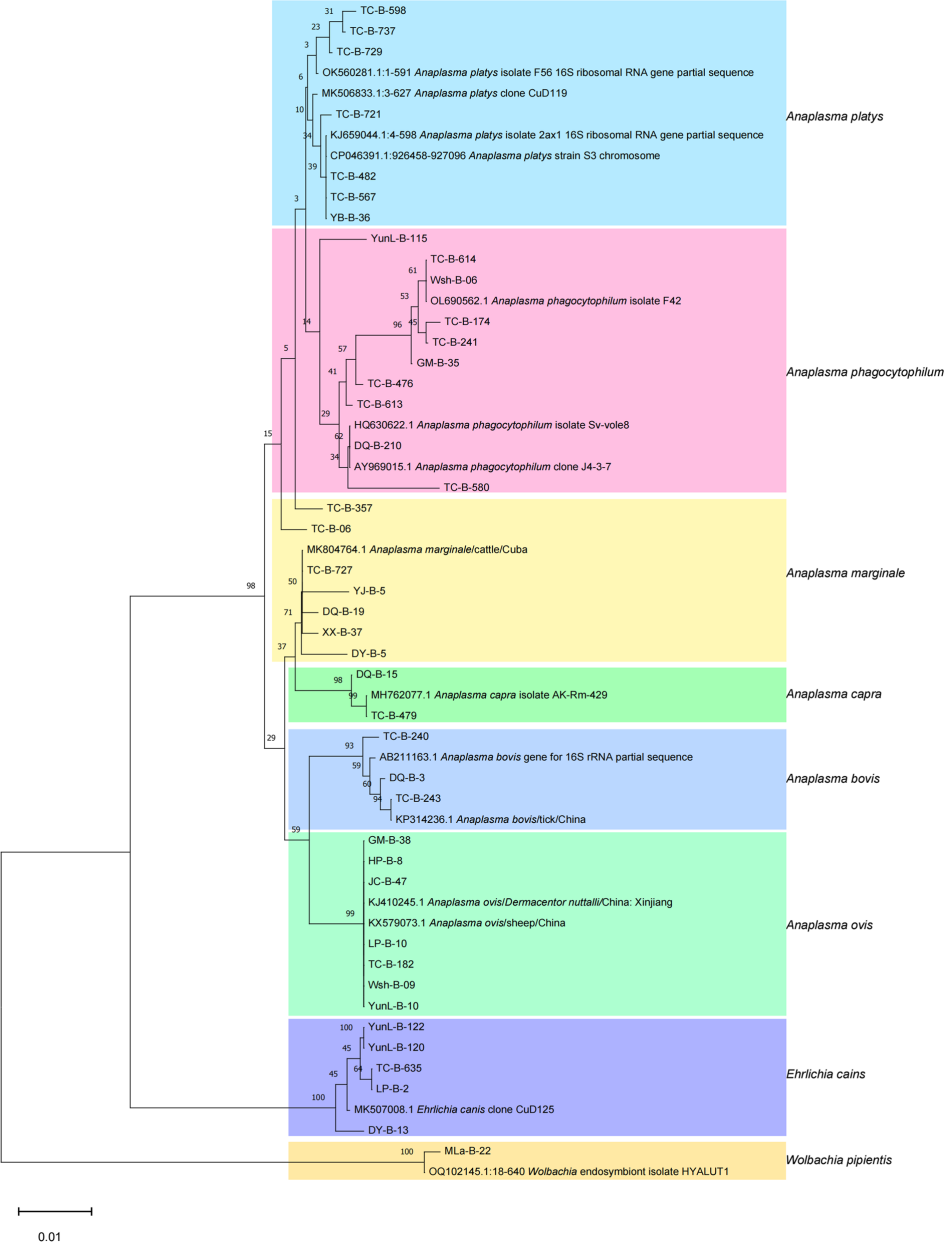
Phylogenetic tree based on partial sequences of 16S rRNA (660 bp) gene of the rickettsiae species derived from livestock. Phylogenetic analysis was performed using the neighbor-joining method and trees were tested by bootstrapping (1000 replicates)

The positivity rate in livestock blood samples collected from 16 counties was 33.68% (800/2375). The highest prevalence was observed in Yingjiang County (81.65%, 89/109), followed by Huaping County (66.02%, 68/103), with significant differences (*P* < 0.001). Among the four types of livestock sampled, *Bovine taurus* exhibited the highest number of infected individuals and the highest prevalence of rickettsial agent infections (45.03%, 317/704, *P* < 0.001). The phylogenetic tree indicated that the rickettsial strains detected in livestock clustered into three distinct groups: *Ehrlichia, Anaplasma*, and *Wolbachia*. The predominant species identified were *A. ovis, A. marginale*, and *A. phagocytophilum*. Notably, *A. phagocytophilum* displayed considerable diversity, with at least 3 variants differing by 1 to 30 base pairs, though they were located within the same clade in the phylogenetic tree (Fig. 5).

### High prevalence and wide distribution of *Rickettsiales* in ticks

The overall positivity rate among 1259 ticks, encompassing 14 species in 5 genera, was 20.65% (260/1259). Rickettsiales agents were observed in all 14 sampling counties, with infestations of 8 out of 14 (57.14%) tick species. The genus *Rhipicephalus* had the highest prevalence (37.07%, 175/472, *P* < 0.001). Specifically, the prevalence of Rickettsiales in the dominant tick species *R. microplus* and *Haemaphysalis montgomeryi* was 36.54% (167/457) and 28.86% (58/201), respectively. *H. yeni* exhibited the highest prevalence (85.71%, 12/14, *P* < 0.001). A total of 13 species of rickettsiae were detected in the tick samples, of which two were identified as pathogenic to humans (Fig. 5). The main tick-associated rickettsial agents were *Candidatus* R. longicornii (*n* = 147) and *A. ovis* (*n* = 67). Among the 14 sampling sites, Jianchuan County had the highest number of positive ticks (*n* = 126), while Heqing County had the highest prevalence (85.71%, 12/14, *P* < 0.001), reflecting their different population sizes. Additionally, ticks collected at altitudes of 2000 to 2499 m had the highest prevalence of rickettsiae (27.08%, 88/325, *P* = 0.004). The prevalence of parasitic ticks (28.47%, 236/829) was significantly higher than that of host-seeking ticks (5.58%, 24/430, *P* < 0.001). Notably, ticks parasitizing *Bos taurus* exhibited the highest prevalence (53.84%, 49/91, *P* < 0.001).

**Fig. 6:**
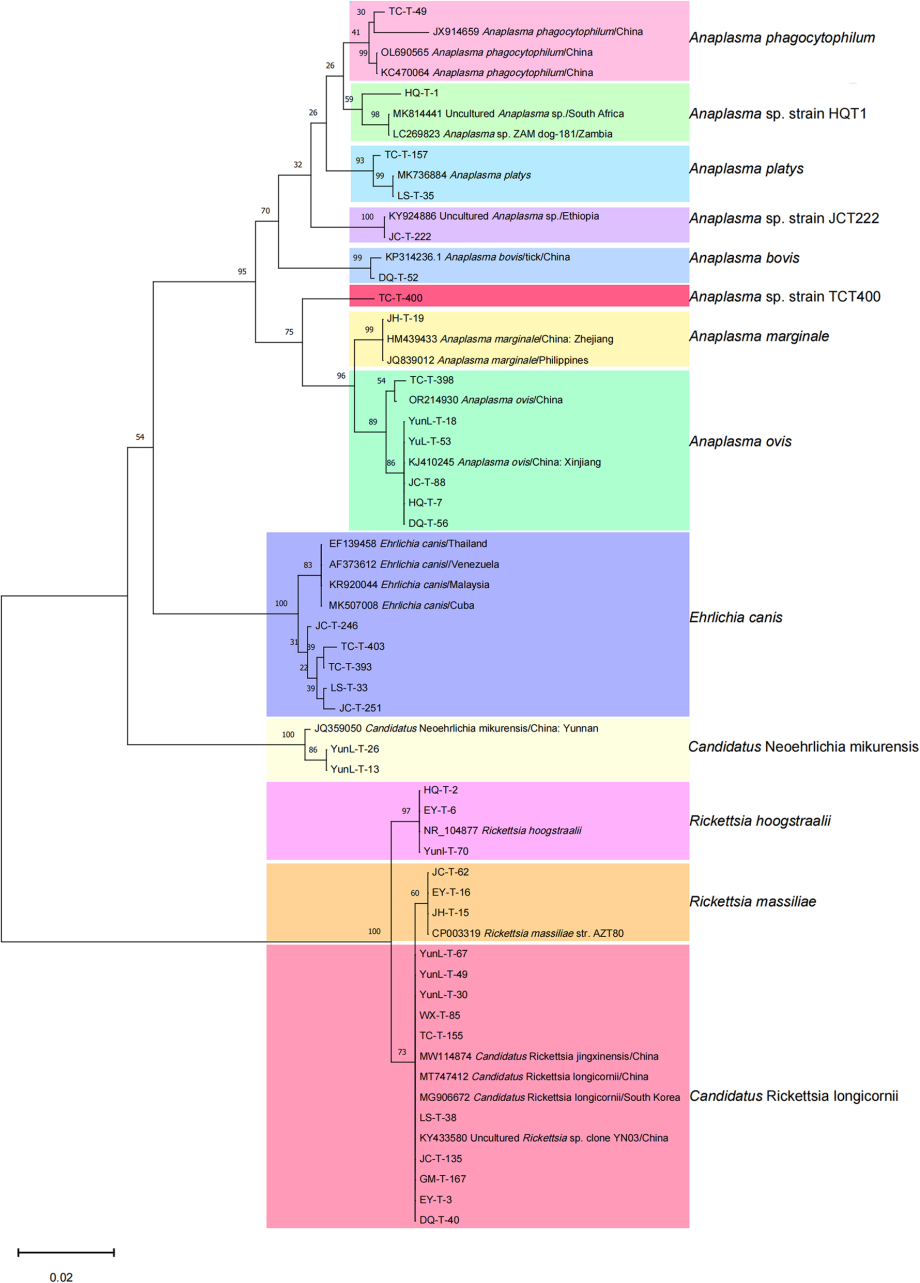
Phylogenetic tree based on partial sequences of 16S rRNA (660 bp) gene of the rickettsiae species derived from ticks. Phylogenetic analysis was performed using the neighbor-joining method and trees were tested by bootstrapping (1000 replicates)

BLAST analysis revealed that the 16S rRNA of HQ-T-1 had 98.49% identity with uncultured *Anaplasma* sp. clone D9_6 (GenBank No. MK814441), JC-T-222 had complete identity with uncultured *Anaplasma* sp. clone Dedessa (GenBank No. KY924886), and TC-T-400 had 99% identity with OL690561, forming a distinct branch in the phylogenetic tree. Combined with phylogenetic analyses, HQ-T-1, JC-T-222, and TC-T-400 were confirmed as monophyletic groups, named *Anaplasma* sp. strain HQT1, *Anaplasma* sp. strain JCT222, and *Anaplasma* sp. strain TCT400, respectively (Fig. 6).

### Characterization of seven pathogenic rickettsial and *Candidatus* Rickettsia longicornii with multigene analysis

**Fig. 7:**
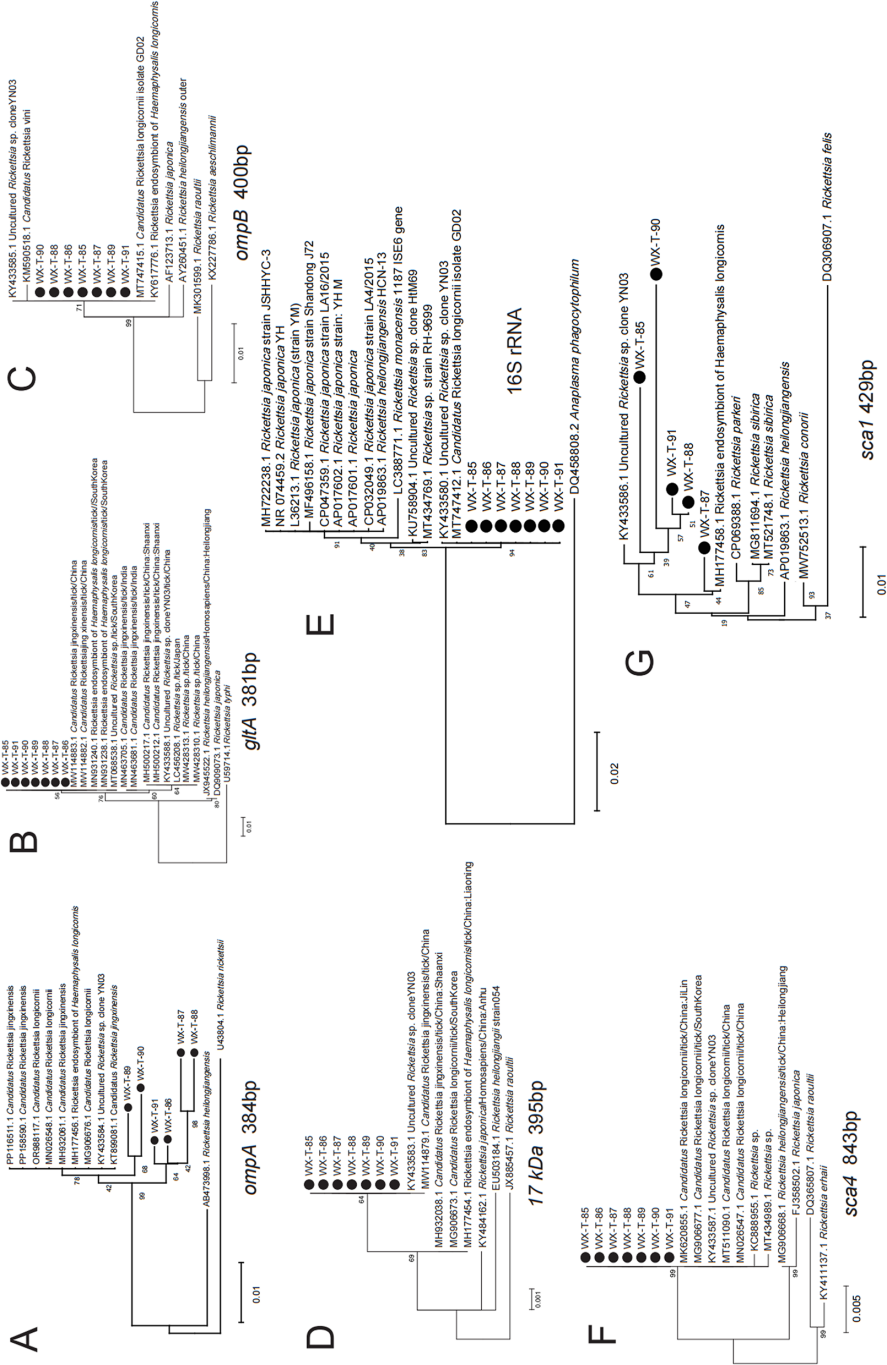
Phylogenetic trees constructed by the MEGA v.6.0 software based on the neighbor-joining method of multigene detected in ticks infected by *Candidatus* Rickettsia longicornii. **A** *ompA* gene (384 bp); **B** *gltA* gene (381 bp); **C** *ompB* gene (400 bp); **D** *17 kDa* gene (395 bp); **E** 16S rRNA gene (nearly full length); **F** *sca4* gene (843 bp); **G** *sca1* gene (429 bp)

Seven tick samples (WX-T-85 to WX-T-91) positive for *Candidatus* R. longicornii from Weixi County were selected for sequencing of the entire *ompA, gltA, ompB, 17 kDa*, 16S rRNA (nearly full length), *sca4*, and *sca1* genes (Fig. 7A-G). The *ompA, ompB*, 16S rRNA, and *sca1* genes displayed 100% homology with the sequences of *Candidatus* R. longicornii or Rickettsia endosymbiont of *Haemaphysalis longicornis.* The *gltA* and *17 kDa* genes from WX-T-90 and WX-T-91 had 100% homology with the sequences of *Candidatus* R. jingxinensis (GenBank Nos. MW114883 and MW114879). The *sca4* gene sequence revealed 99.51% homology with *Candidatus* R. longicornii (GenBank Nos. MK620855, MG906677). Despite some conflicting sequences, the rickettsial organism is likely *Candidatus* R. longicornii.

Both the full-length 16S rRNA gene (Fig. S1A) and the *groEL* gene of CNM (Fig. S1B) detected in this study were remarkably distinct from other sequences detected in China (Heilongjiang), Germany, and Switzerland. Among the three clusters of CNM based on the *groEL* gene the phylogenetic tree indicated that the present sequences belong to cluster I (southwest) and cluster III (southeast) [37] (Fig. S1B). Although the sequences from Lushui and Weixi were close to the southwest branch (I), they clustered into small branches, indicating they differ from previous ones in Yunnan.

Five *Anaplasma*-associated pathogens (*A. capra, A. ovis, A. bovis, A. marginale,* and *A. phagocytophilum*) were confirmed in samples. The *groEL* (Fig. S2B) and *gltA* (Fig. S2C) genes of *A. capra* revealed 100% homology between *Capra aegagrus* in Tengchong and patients in Heilongjiang [8]. The *msp4* gene (Fig. S2D) of *A. capra* had 99.8% homology, indicating pronounced similarity with the pathogen infecting patients. Variations of *A. capra* were also detected in Tengchong, Deqin, and Shangri-La (Fig. S2). The phylogenetic tree based on the msp4 and entire 16S rRNA gene (Fig. S3A) of *A. ovis* formed a cohesive branch closely aligned with reference sequences. Sequences detected in Yunlong consistently clustered within a separate branch, demonstrating a close genetic relationship across different hosts within this geographic region. The *gltA* gene (Fig. S3C) of *A. ovis* distributed into two small branches. The *groEL* gene of *A. bovis*, *A. marginale*, and *A. phagocytophilum* also indicated diversity and distinctive distribution among their host ticks and animals (Fig. S4). The *groEL* and *gltA* genes of *E. canis* clustered in a distinct branch along with other sequences identified in ticks in China, with sister branches from Thailand, the Philippines, Spain, and France. The *Dsb* and *TRP36* genes of *E. canis* formed unique, singular clades (Fig. S5).

## Discussion

We identified the co-circulation of multiple species of Rickettsiales bacteria, including previously uncharacterized species, among small mammals, livestock, and ticks in Yunnan Province. This underscores the enduring importance of Rickettsiales infections in southwestern China and the need for ongoing surveillance of local arthropods, mammals, and humans.

The extensive diversity of tick-associated *Rickettsia* species globally is well documented, with instances such as *R. tillamookensis* in the United States and various species found in wild mammals in Morocco and Mauritania [38, 39]. Advances in phylogenetics and molecular detection techniques have greatly facilitated the identification of numerous novel Rickettsiales species associated with mammals and arthropods [5]. In China, new and uncharacterized rickettsial species continue to be discovered such as *Rickettsia* sp. sw. in Yunnan Province and *Ehrlichia* sp. in Inner Mongolia [23, 40]. Recent reports indicate a high diversity of *Rickettsia* spp. and *Ehrlichia* spp. in five species of tick from Yunnan Province [26]. We further identified six novel strains and one new record in Yunnan along with known Rickettsiales agents, emphasizing the remarkable diversity of Rickettsiales bacteria in ticks, as well as in small mammals and livestock.

There are variations in the reported prevalence of rickettsial agent infections among wild small mammals in different regions such as Southwest China and the Indo-China peninsula (2.02%), Slovakia (19.10%), and Kazakhstan (2.72%) [41–43]. In our study, the genus *Dremomys* exhibited a significantly high rate of Rickettsiales positivity among small mammals, while the highest frequency was observed in the genus *Rattus*, which is widely distributed and commonly found near local residences. Therefore, these two genera should be focused on when monitoring rickettsial infections. Additionally, our observations indicated changes in rickettsial prevalence based on sex and eco-habitats. The higher infection rates in males may be attributed to their more competitive and aggressive behaviors, leading to increased contact and susceptibility to infection. Furthermore, samples collected from alpine meadows revealed higher prevalence rates of Rickettsiales agents, possibly due to the presence of dominant host animals in these habitats. These observations warrant further detailed investigation. In summary, the rickettsial agents detected in these small mammals exhibited distinctive genetic diversity characteristics both interspecifically and intraspecifically.

Moreover, diverse rickettsial species, such as *Rickettsia* spp., *Anaplasma* spp., *Ehrlichia* spp., *Neoehrlichia* spp., and *Wolbachia* spp., were identified in 33.68% (800/2375) of livestock and were distributed across almost all surveyed counties. Given the close contact between humans and livestock, this highlights a potentially high risk of human exposure to these rickettsial agents. Among the rickettsial agents detected in livestock, *A. phagocytophilum, A. capra, A. platys*, and A. *bovis* variant were confirmed to possess human pathogenicity in earlier studies [7, 8, 44, 45]. Additionally, *A. bovis* and *A. bovis*-like infections have recently been identified in patients from China and the United States [16, 46]. Conversely, *A. marginale* and *E. canis* are recognized as pathogenic to animals rather than humans [47, 48].

Numerous reports in previous literatures have highlighted the risk posed by Rickettsiales, such as *A. ovis* infections in Iran and *Anaplasma* spp. in Pakistan [49, 50]. Our large-scale study confirmed that locations in Yuanmou County, areas at latitudes over 4000 m, and alpine meadow regions exhibited significantly higher Rickettsiales positivity rates among small mammals. Additionally, *A. phagocytophilum* was frequently detected in small mammals, livestock, and ticks, confirming its widespread distribution in Yunnan Province, particularly in Deqin County, where there is a higher potential for forming transmission chains.

Certain rickettsial species may exhibit specific host or vector associations, indicating host tropism for these agents. While some Rickettsiales are host-specific, others switch hosts or circulate regularly among different hosts, particularly mammals and blood-sucking arthropods [5]. Since most emerging human infectious diseases originate from spillover events from vectors or animal hosts, assessing infections among hosts, vectors, and febrile patients in local representative areas is crucial for understanding tick-associated Rickettsiales [4, 6, 7]. Therefore, the characterization and identification of novel Rickettsiales are of significant importance for both human and animal health.

Multigene analysis of *E. canis* from representative samples revealed a clustering pattern, distinct from international strains, indicating that the *E. canis* detected in Yunnan Province has unique genetic characteristics. Similarly, the multigene analysis of CNM from Weixi samples revealed close genetic similarities to those from southeastern China, yet notable divergence from strains previously detected in Yunnan, Heilongjiang, and abroad. Based on the previous classification standards for CNM, the CNM from Weixi should be considered distinct variants [44]. Our results also indicated that *A. ovis* was the most prevalent species, with a high positivity rate, widespread distribution, and interspecific differences in the survey region. However, sequences from Yunlong consistently clustered within a separate branch, demonstrating close genetic relationships across different hosts within this specific geographic region.

A comprehensive analysis of multiple genes for our *Rickettsia* agent, which was closely related to *Candidatus* R. longicornii, *Rickettsia* YN03, and *R. jingxinensis*, revealed that the nomenclature of “*Candidatus* R. jingxinensis” is not rigorous [51–54]. In fact, “*Candidatus* R. jingxinensis” and “*Candidatus* R. longicornii” should be considered synonyms. Based on the rules of species nomenclature, the name “*Candidatus* R. longicornii” should be accepted as valid, hence it should be referred to as *R. longicornii*. Similarly, the name *Rickettsia* YN03 should also be synonymized as *R. longicornii*, despite the observed degree of variation and considerable diversity.

Our primary limitation was the absence of human case records and samples, which precluded the confirmation of potential infections among local residents. Most study areas are located in remote mountainous regions of Yunnan Province, characterized by a high population of ethnic minorities. These areas are economically underdeveloped, and the local population generally has low awareness of self-protection measures and limited access to medical services. Furthermore, medical services at the grassroots level are inadequate, lacking the capacity for clinical diagnosis and management of emerging tick-associated rickettsioses, leading to frequent missed or misdiagnosed cases. Additionally, the prevalence of tick-associated rickettsioses could negatively impact ecotourism and economic development in Yunnan Province. Therefore, early surveillance of these emerging Rickettsiales among hosts, vectors, and exposed populations in these hotspots is crucial for effective prevention and control.

## Conclusions

This large-scale study underscores the genetic diversity and overall prevalence of emerging tick-associated Rickettsiales within biodiversity hotspots in Yunnan Province. These findings reveal the substantial threats posed by emergent Rickettsiales and serve as a crucial reference for the prevention and control of related zoonotic diseases in southwestern China. Given the impact of these emerging Rickettsiales in these hotspots, urgent surveillance and implementation of prevention and control measures for the local human populations is warranted.

### Supplementary Information

Supplementary Material 1: Supplementary Table 1. Primers used for multigene testing. Supplementary Table 2. Overall prevalence of animals and vectors from different species with emerging Rickettsiales in Yunnan Province. Supplementary Table 3. The diversity of BLAST-based sequence analysis of tick associated Rickettsiales in animals and vectors. Supplementary Fig. 1. Phylogenetic trees constructed by the MEGA v.6.0 software based on the neighbor-joining method of multigene detected in small mammals infected by *Candidatus* Neoehrlichia mikurensis. A: 16S rRNA gene(1550 bp); B:* groEL* gene (891 bp). Supplementary Fig. 2. Phylogenetic trees constructed by the MEGA v.6.0 software based on the neighbor-joining method of multigene detected in livestock infected by *Anaplasma capra*. A: 16S rRNA gene (660 bp); B: *groEL* gene (1008 bp); C: *gltA* gene (636 bp); D: *msp4* gene (613 bp). Supplementary Fig. 3. Phylogenetic trees constructed by the MEGA v.6.0 software based on the neighbor-joining method of multigene detected in different samples infected *Anaplasma ovis*. Triangles, circles and rectangles represent livestock, small mammals, and ticks in this study respectively. A: 16S rRNA gene (1850 bp); B:* groEL* gene (2066 bp); C: *gltA* gene (792 bp); D: *msp4* gene (597 bp). Supplementary Fig. 4. Phylogenetic trees constructed by the MEGA v.6.0 software based on the neighbor-joining method of *groEL* gene (372 bp) detected in different samples. Triangles, circles and rectangles represent livestock, small mammals, and ticks in this study respectively. Supplementary Fig. 5. Phylogenetic trees constructed by the MEGA v.6.0 software based on the neighbor-joining method of multigene detected in different samples infected *Ehrlichia canis*. Circles and rectangles represent small mammals and ticks in this study respectively. A: *Dsb* gene (409 bp); B: *gltA* gene (125 bp); C: *TRP36* gene (800–1000 bp); D: *groEL *gene (364 bp).

## Data Availability

Sequencing data generated during study was submitted to China National Microbiology Data Center under the BioProject. For each biological sample, the accession number as well as the corresponding link can be found in Supplementary Table.
