# Supplementary material for: Genetic diversity and prevalence of emerging Rickettsiales in Yunnan Province: a large-scale study
Source: Infect Dis Poverty. 2024 Jul 10;13:54. doi: 10.1186/s40249-024-01213-4 (PMC11234784; doi:10.1186/s40249-024-01213-4)
Supplement: Supplementary file 1 — Supplementary Material 1: Supplementary Table 1. Primers used for multigene testing. Supplementary Table 2. Overall prevalence of animals and vectors from different species with emerging Rickettsiales in Yunnan Province. Supplementary Table 3. The diversity of BLAST-based sequence analysis of tick associated Rickettsiales in animals and vectors. Supplementary Fig. 1. Phylogenetic trees constructed by the MEGA v.6.0 software based on the neighbor-joining method of multigene detected in small mammals infected by Candidatus Neoehrlichia mikurensis. A: 16S rRNA gene(1550 bp); B: groEL gene (891 bp). Supplementary Fig. 2. Phylogenetic trees constructed by the MEGA v.6.0 software based on the neighbor-joining method of multigene detected in livestock infected by Anaplasma capra. A: 16S rRNA gene (660 bp); B: groEL gene (1008 bp); C: gltA gene (636 bp); D: msp4 gene (613 bp). Supplementary Fig. 3. Phylogenetic trees constructed by the MEGA v.6.0 software based on the neighbor-joining method of multigene detected in different samples infected Anaplasma ovis. Triangles, circles and rectangles represent livestock, small mammals, and ticks in this study respectively. A: 16S rRNA gene (1850 bp); B: groEL gene (2066 bp); C: gltA gene (792 bp); D: msp4 gene (597 bp). Supplementary Fig. 4. Phylogenetic trees constructed by the MEGA v.6.0 software based on the neighbor-joining method of groEL gene (372 bp) detected in different samples. Triangles, circles and rectangles represent livestock, small mammals, and ticks in this study respectively. Supplementary Fig. 5. Phylogenetic trees constructed by the MEGA v.6.0 software based on the neighbor-joining method of multigene detected in different samples infected Ehrlichia canis. Circles and rectangles represent small mammals and ticks in this study respectively. A: Dsb gene (409 bp); B: gltA gene (125 bp); C: TRP36 gene (800–1000 bp); D: groEL gene (364 bp). [file 40249_2024_1213_MOESM1_ESM.docx]

Table S1. Primers used for multigene testing

| Specimens | Target gene | Primer name | Primer sequence (5’→3’) |
| --- | --- | --- | --- |
| Rattus species | COI | BatL5310 | CCTACTCRGCCATTTTACCTATG |
|  |  | R6036R | ACTTCTGGGTGTCCAAAGAATCA |
| Tick species | COI | T-COIF | GAGTCGGTAAAATGGCGCTAC |
|  |  | T-COIR | GCTATCTTTAAGAGGGTAATA |
| Candidatus Rickettsia longicornii | 16S rRNA | Eh-out1 | TTGAGAGTTTGATCCTGGCTCAGAACG |
|  |  | Eh-out2U | CACCTCTACACTAGGAATTCCACTATC |
|  |  | 3-17U | WAAGGWGGTAATCCAGC |
|  |  | Eh-out2fU | GATAGTGGAATTCCTAGTGTAGAGGTG |
|  | gltA | CS2d | ATGACCAATGAAAATAATAAT |
|  |  | CSEndr | CTTATACTCTCTATGTACA |
|  |  | RpCS877f | GGGGACCTGCTCACGGCGG |
|  |  | RpCS1258r | ATTGCAAAAAGTACAGTGAACA |
|  | ompA | Rr190.70 | ATGGCGAATATTTCTCCAAAA |
|  |  | Rr190.602n | AGTGCAGCATTCGCTCCCCCT |
|  |  | Tara38s1 | AAAACCGCTTTATTACC |
|  |  | Tara384r1 | GGCAACAAGTTACCTCCT |
|  | ompB | rompB OF | GTAACCGGAAGTAATCGTTTCGTAA |
|  |  | rompB OR | GCTTTATAACCAGCTAAACCACC |
|  |  | rompB SFG IF | GTTTAATACGTGCTGCTAACCAA |
|  |  | rompB SFG/TG IR | GGTTTGGCCCATATACCATAAG |
|  | 17kDa | 17k3 | GCTTTACAAAATTCTAAAAACCATATA |
|  |  | 17k5 | TGTCTATCAATTCACAACTTGCC |
|  |  | Tara17KD 13s1 | ATTGTCCGTCAGGTTGGC |
|  |  | Tara17KD 408r1 | CGGGCGGTATGAATAAGC |
|  | sca1 | sca159 | CCCGTCTCGTGACTTACC |
|  |  | sca936 | AGCATTAGGCGATGGTAG |
|  |  | sca183 | TAAGCCGATATTTGGAGT |
|  |  | sca612 | TTTATGGATGCGTGGTAT |
|  | sca4 | sc4-1 | ATGTCTCTGAATTAAGCAATGC |
|  |  | Rj2837r | CCTGATACTACCCTTACATC |
|  |  | sc4-3 | AATTATTAGGCTCTGTATTAAAGA |
|  |  | sc4-4 | GAAAGGATAGCACGAAAAGTA |
| Candidatus Neoehrlichia mikurensis | 16S rRNA | Eh-out1 | TTGAGAGTTTGATCCTGGCTCAGAACG |
|  |  | Eh-out2U | CACCTCTACACTAGGAATTCCACTATC |
|  |  | 3-17U | WAAGGWGGTAATCCAGC |
|  |  | Eh-out2fU | GATAGTGGAATTCCTAGTGTAGAG GTG |
|  | groEL | CNM-out1 | TGGCAAATGTAGTTGTAACAGG |
|  |  | CNM-out2 | TCTACTTCACTTGAACCGCCA |
|  |  | CNM-in1 | GCTATTAGTAAGCCTTATGGTAC |
|  |  | CNM-in2 | GAAGAATTACTATCTACGCTACC |
| *Anaplasma capra* | groEL | groe1F | ATGGCGAATGTTGTTGTT |
|  |  | groe1472r | TTAGCGTAGTTCATGGTGTC |
|  |  | groe3F | GGCGAATGTTGTTGTTACGG |
|  |  | groe1011r | SACRCTTCCTATGATTGTGGT |
|  | msp4 | Acm41f | CAGTCTGCGCCTGCTCCCTAC |
|  |  | Acm4799r | AGGAATCTTGCTCCAAGGTTA |
|  |  | Acm480f | CGGAGTCATGGGAGGTAG |
|  |  | Acm4693r | AGGATTCGTCAAAGAGCC |
|  | gltA | AcgA122f | GCGTTGCTCATCTCCTGT |
|  |  | ACgA790r | GATCGTAATTCTTGTAGACCCT |
|  |  | ACgA122f | GCGTTGCTCATCTCCTGT |
|  |  | ACgA758r | CCCATAAGCCTCACTCCAT |
| A. ovis | 16S rRNA | rp2 | ACGGCTACCTTGTTACGACTT |
|  |  | HER16SD | GGTACCYACAGAAGAAGTCC |
|  |  | fD1 | AGAGTTTGATCCTGGCTCAG |
|  |  | HER16SR | TAGCACTCATCGTTTACAGC |
|  | groEL | ovisge37f | AAATCTATAAGGGAGGTAGTGC |
|  |  | ovisg1048r | GGCTAGTCCTGCTGGTAAT |
|  |  | ovisgr419f | AGGACGAAATTGCACAGG |
|  |  | ovisg1474r | CGTTAGCGTAGTTCATGGTG |
|  | msp4 | Aomp4-63f | CCTGCTCCCTACTTGTTA |
|  |  | Aomp4-769r | GTTATGTGCGGGTATGTC |
|  |  | Aomp4-168f | CTTACAGCCCAGCGTTTC |
|  |  | Aomp4-765r | TGTGCGGGTATGTCCTTG |
|  | gltA | 23F | GCGATTTTAGAGTGYGGAGATTG |
|  |  | 1104R | TACAATACCGGAGTAAAAGTCAA |
|  |  | 148F | GGGTTCMTGTCYACTGCTGCGTG |
|  |  | 940R | TTGGATCGTARTTCTTGTAGACC |
| *A. phagocytophilum* | groEL | ANA-GroF | TCATTACTCAGAGTGCTTCTCAGTG |
|  |  | ANA-GroR | CGATCAAACTGCATACCATCAGTC |
| Ehrlichia cains | groEL | gro607F | GAAGATGCWGTWGGWTGTACKGC |
|  |  | gro1294R | AGMGCTTCWCCTTCWACRTCYTC |
|  |  | gro677F | ATTACTCAGAGTGCTTCTCARTG |
|  |  | gro1121R | TGCATACCRTCAGTYTTTTCAAC |
|  | gltA | CAN-M61F | TTATCTGTTTATGTTATATAAGC |
|  |  | CAN1317R | CAGTACCTATGCATATCAATCC |
|  | Dsb | Dsb-330 | GATGATGTCTGAAGATATGAAACA AAT |
|  |  | Dsb-728 | CTGCTCGTCTATTTTACTTCTTAAA GT |
|  | TRP36 | TRP36-F2 | TTTAAAACAAAATTAACACACTA |
|  |  | TRP36-R1 | AAGATTAACTTAATACTCAATATT ACT |
|  |  | TRP36-R1 | AAGATTAACTTAATACTCAATATT ACT |
|  |  | TRP36-DF | CACACTAAAATGTATAATAAAGC |

Table S2. Overall prevalence of animals and vectors from different species with emerging Rickettsiales in Yunnan province

| Species | No. of test | No. of positive | Prevalence (%) | Species | No. of test | No. of positive | Prevalence (%) |
| --- | --- | --- | --- | --- | --- | --- | --- |
| **Small mammals** | | | | *Leopoldamys edwardsi* | 13 | 0 | 0.00% |
| *Rattus tanezumi* | 720 | 22 | 3.06 | *Apodemus agrarius* | 12 | 0 | 0.00 |
| *Apodemus draco* | 452 | 14 | 3.10 | *Berylmys bowersi* | 11 | 0 | 0.00 |
| *Apodemus chevrieri* | 429 | 2 | 0.47 | *Niviventer excelsior* | 11 | 0 | 0.00 |
| *Rattus andamanensis* | 272 | 21 | 7.72 | *Vernaya fulva* | 9 | 0 | 0.00 |
| *Niviventer confucianus* | 187 | 4 | 2.14 | *Eothenomys proditor* | 7 | 0 | 0.00 |
| *Eothenomys miletus* | 172 | 2 | 1.16 | *Rattus pyctoris* | 7 | 0 | 0.00 |
| *Unknow species* | 165 | 0 | 0.00 | *Eothenomys melanogaster* | 5 | 0 | 0.00 |
| *Suncus murinus* | 124 | 2 | 1.61 | *Ochotona forresti* | 5 | 0 | 0.00 |
| *Mus pahari* | 123 | 1 | 0.81 | *Sorex unguiculatus* | 5 | 0 | 0.00 |
| *Apodemus latronum* | 121 | 4 | 3.31 | *Suncus etruscus* | 5 | 0 | 0.00 |
| *Niviventer fulvescens* | 100 | 0 | 0.00 | *Tamiops swinhoei* | 5 | 0 | 0.00 |
| *Rattus norvegicus* | 97 | 5 | 5.15 | *Scaptonyx fusicaudus* | 3 | 1 | 33.33 |
| *Anourosorex squamipes* | 84 | 0 | 0.00 | *Bandicota indica* | 2 | 0 | 0.00 |
| *Episoriculus caudatus* | 83 | 6 | 7.23 | *Eozapus setchuanus* | 2 | 0 | 0.00 |
| *Mus caroli* | 77 | 0 | 0.00 | *Crocidura lasiura* | 1 | 0 | 0.00 |
| *Crocidura dracula* | 73 | 1 | 1.37 | *Dremomys rufigenis* | 1 | 0 | 0.00 |
| *Eothenomys eleusis* | 73 | 4 | 5.48 | *Niviventer brahma* | 1 | 0 | 0.00 |
| *Tupaia belangeri* | 71 | 0 | 0.00 | *Typhlomys cinereus* | 1 | 0 | 0.00 |
| *Crocidura attenuata* | 69 | 0 | 0.00 | **Livestock** | | | |
| *Rattus nitidus* | 69 | 3 | 4.35 | Sheep | 1107 | 446 | 40.29 |
| *Eothenomys custos* | 68 | 4 | 5.88 | Cow | 704 | 317 | 45.03 |
| *Ochotona thibetana* | 65 | 3 | 4.62 | Dog | 559 | 37 | 6.62 |
| *Episoriculus leucops* | 62 | 0 | 0.00 | Cat | 5 | 0 | 0.00 |
| *Sorex cylindricauda* | 48 | 2 | 4.17 | **Ticks** | | | |
| *Neodon leucurus* | 47 | 2 | 4.26 | *Rhipicephalus microplus* | 457 | 167 | 36.54 |
| *Crocidura vorax* | 39 | 0 | 0.00 | *Ixodes ovatus* | 302 | 8 | 2.65 |
| *Micromys minutus* | 33 | 0 | 0.00 | *Haemaphysalis montgomeryi* | 201 | 58 | 28.86 |
| *Uropsilus aequodonenia* | 33 | 0 | 0.00 | *Haemaphysalis Kolonini* | 163 | 2 | 1.23 |
| *Eothenomys cachinus* | 32 | 0 | 0.00 | *Ixodes granulatus* | 51 |  | 0.00 |
| *Neotetracus sinensis* | 30 | 0 | 0.00 | *Rhipicephalus haemaphysaloides* | 15 | 8 | 53.33 |
| *Niviventer eha* | 30 | 0 | 0.00 | *Hae.* spp. | 14 |  | 0.00 |
| *Sorex asper* | 30 | 1 | 3.33 | *Haemaphysalis yeni* | 14 | 12 | 85.71 |
| *Neodon clarkei* | 28 | 0 | 0.00 | *Haemaphysalis menglaensis* | 10 |  | 0.00 |
| *Hylomys suillus* | 27 | 0 | 0.00 | *Ixodes acutitarsus* | 10 | 3 | 30.00 |
| *Niviventer andersoni* | 24 | 1 | 4.17 | *Haemaphysalis nepalensis* | 9 |  | 0.00 |
| *Eothenomys olitor* | 19 | 1 | 5.26 | *Dermacentor auratus* | 7 |  | 0.00 |
| *Mus musculus* | 18 | 0 | 0.00 | *Haemaphysalis longicornis* | 5 | 2 | 40.00 |
| *Dremomys pernyi* | 16 | 1 | 6.25 | *Amblyomma testudinarium* | 1 |  | 0.00 |
| *Crocidura indochinensis* | 14 | 0 | 0.00 |  |  |  |  |

Table S3. The diversity of BLAST-based sequence analysis of tick-associated Rickettsiales in animals and vectors

| Sample number | BLAST result | Accession | Query coverage（%） | Percent identity （%） |
| --- | --- | --- | --- | --- |
| SP-030 | *Candidatus* Neoehrlichia mikurensis | [HM439431.3](https://www.ncbi.nlm.nih.gov/nucleotide/HM439431.3?report=genbank&log$=nucltop&blast_rank=1&RID=VEV269BD013) | 99.00 | 99.84 |
| YM-159 | *Candidatus* Neoehrlichia mikurensis | [HM439431.3](https://www.ncbi.nlm.nih.gov/nucleotide/HM439431.3?report=genbank&log$=nucltop&blast_rank=1&RID=VEV269BD013) | 99.00 | 99.84 |
| NE-038 | *Candidatus* Neoehrlichia mikurensis | [HM439431.3](https://www.ncbi.nlm.nih.gov/nucleotide/HM439431.3?report=genbank&log$=nucltop&blast_rank=1&RID=VEV269BD013) | 99.00 | 99.84 |
| JG-061 | *Candidatus* Neoehrlichia mikurensis | [HM439431.3](https://www.ncbi.nlm.nih.gov/nucleotide/HM439431.3?report=genbank&log$=nucltop&blast_rank=1&RID=VEV269BD013) | 99.00 | 99.84 |
| LC-146 | *Candidatus* Neoehrlichia mikurensis | [HM439431.3](https://www.ncbi.nlm.nih.gov/nucleotide/HM439431.3?report=genbank&log$=nucltop&blast_rank=1&RID=VEV269BD013) | 99.00 | 99.68 |
| MLian-033 | *Candidatus* Neoehrlichia mikurensis | HM439431.3 | 100.00 | 99.68 |
| TC-024 | *Candidatus* Neoehrlichia mikurensis | HM439431.3 | 100.00 | 99.68 |
| YJ-014 | *Candidatus* Neoehrlichia mikurensis | [OK560170.1](https://www.ncbi.nlm.nih.gov/nucleotide/OK560170.1?report=genbank&log$=nucltop&blast_rank=1&RID=WT0VZ1FM016) | 100.00 | 99.48 |
| DQ-179 | *Candidatus* Neoehrlichia mikurensis | [AB196304.1](https://www.ncbi.nlm.nih.gov/nucleotide/AB196304.1?report=genbank&log$=nucltop&blast_rank=1&RID=WT0WEYUG016) | 100.00 | 99.65 |
| WX-183 | *Candidatus* Neoehrlichia mikurensis | GU227699.1 | 100.00 | 99.39 |
| YunL-065 | *Candidatus* Neoehrlichia mikurensis | [AB196304.1](https://www.ncbi.nlm.nih.gov/nucleotide/AB196304.1?report=genbank&log$=nucltop&blast_rank=1&RID=WT0WEYUG016) | 100.00 | 99.29 |
| MLian-240 | *Ehrlichia sp. YN04* | KY433582.1 | 96.00 | 99.38 |
| YJ-005 | *Ehrlichia sp. YN04* | KY433582.1 | 100.00 | 100.00 |
| YM-090 | *Anaplasma ovis* | [KX579073.1](https://www.ncbi.nlm.nih.gov/nucleotide/KX579073.1?report=genbank&log$=nucltop&blast_rank=1&RID=VEW62WR6016) | 100.00 | 100.00 |
| YM-249 | *Anaplasma ovis* | [KX579073.1](https://www.ncbi.nlm.nih.gov/nucleotide/KX579073.1?report=genbank&log$=nucltop&blast_rank=1&RID=VEW62WR6016) | 100.00 | 99.04 |
| MLian-036 | *Anaplasma ovis* | [OQ909500.1](https://www.ncbi.nlm.nih.gov/nucleotide/OK560163.1?report=genbank&log$=nuclalign&blast_rank=2&RID=VEX1Z0MZ016) | 100.00 | 98.23 |
| DQ-098 | *Anaplasma* sp. strain DQ098 | KU189193.1 | 100.00 | 97.48 |
| DQ-292 | *Anaplasma* sp. strain DQ098 | KU189193.1 | 100.00 | 97.48 |
| SP-036 | *A. phagocytophilum* | [CP015376.1](https://www.ncbi.nlm.nih.gov/nucleotide/CP015376.1?report=genbank&log$=nucltop&blast_rank=1&RID=VEY4W7EV013) | 91.00 | 99.09 |
| TC-013 | *A. phagocytophilum* | OL690565.1 | 100.00 | 99.43 |
| YJ-316 | *A. phagocytophilum* | FJ788512.1 | 99.00 | 99.81 |
| YM-063 | *A. phagocytophilum* | KC470064.1 | 100.00 | 99.50 |
| YM-103 | *A. phagocytophilum* | KC470064.1 | 100.00 | 99.50 |
| YL-020 | *Wolbachia* sp. strain YL020 | OX366385.1 | 100.00 | 99.68 |
| XX-329 | *Wolbachia* sp. strain YM011 | [DQ115537.1](https://www.ncbi.nlm.nih.gov/nucleotide/DQ115537.1?report=genbank&log$=nucltop&blast_rank=2&RID=WT4HJPXW013) | 100.00 | 98.83 |
| MLian-056 | *Wolbachia* sp. strain YM011 | [DQ115537.1](https://www.ncbi.nlm.nih.gov/nucleotide/DQ115537.1?report=genbank&log$=nucltop&blast_rank=2&RID=WT4HJPXW013) | 100.00 | 98.99 |
| YM-011 | *Wolbachia* sp. strain YM011 | [CP116767.1](https://www.ncbi.nlm.nih.gov/nucleotide/CP116767.1?report=genbank&log$=nucltop&blast_rank=1&RID=VEYV5M07016) | 100.00 | 98.72 |
| LC-126 | *Wolbachia* sp. strain YM011 | [DQ115537.1](https://www.ncbi.nlm.nih.gov/nucleotide/DQ115537.1?report=genbank&log$=nucltop&blast_rank=2&RID=WT4HJPXW013) | 100.00 | 99.66 |
| MZ-112 | *Wolbachia* sp. strain YM011 | [DQ115537.1](https://www.ncbi.nlm.nih.gov/nucleotide/DQ115537.1?report=genbank&log$=nucltop&blast_rank=2&RID=WT4HJPXW013) | 100.00 | 99.50 |
| YS-016 | *Wolbachia* sp. strain YM011 | [DQ115537.1](https://www.ncbi.nlm.nih.gov/nucleotide/DQ115537.1?report=genbank&log$=nucltop&blast_rank=2&RID=WT4HJPXW013) | 100.00 | 99.50 |
| JG-008 | *Wolbachia* sp. strain YM011 | [DQ115537.1](https://www.ncbi.nlm.nih.gov/nucleotide/DQ115537.1?report=genbank&log$=nucltop&blast_rank=2&RID=WT4HJPXW013) | 100.00 | 99.50 |
| JG-013 | *Wolbachia* sp. strain YM011 | [DQ115537.1](https://www.ncbi.nlm.nih.gov/nucleotide/DQ115537.1?report=genbank&log$=nucltop&blast_rank=2&RID=WT4HJPXW013) | 100.00 | 99.50 |
| YM-174 | *Rickettsia typhi* | NR_074394.1 | 100.00 | 99.52 |
| LC-024 | *Candidatus R. longicornii* | MT747412.1 | 100.00 | 99.38 |
| TC-B-06 | *A. marginale* | MN187218.1 | 100.00 | 99.16 |
| TC-B-357 | *A. marginale* | [OL660546.1](https://www.ncbi.nlm.nih.gov/nucleotide/OL660546.1?report=genbank&log$=nucltop&blast_rank=1&RID=WT7X8JXK016) | 100.00 | 98.99 |
| YunL-B-115 | *A. phagocytophilum* | [CP035303.1](https://www.ncbi.nlm.nih.gov/nucleotide/CP035303.1?report=genbank&log$=nucltop&blast_rank=34&RID=WT7XN1AB013) | 100.00 | 97.65 |
| TC-B-580 | *A. phagocytophilum* | OL690558.1 | 93.00 | 98.39 |
| DQ-B-210 | *A. phagocytophilum* | MK814412.1 | 100.00 | 99.65 |
| TC-B-613 | *A. phagocytophilum* | OL690562.1 | 99.00 | 98.31 |
| TC-B-476 | *A. phagocytophilum* | CP015376.1 | 99.00 | 97.82 |
| GM-B-35 | *A. phagocytophilum* | OL690562.1 | 99.00 | 98.98 |
| TC-B-241 | *A. phagocytophilum* | OL690562.1 | 100.00 | 99.06 |
| TC-B-174 | *A. phagocytophilum* | OL690562.1 | 100.00 | 99.46 |
| Wsh-B-06 | *A. phagocytophilum* | [OL690562.1](https://www.ncbi.nlm.nih.gov/nucleotide/OL690562.1?report=genbank&log$=nucltop&blast_rank=3&RID=WTNHZ8ZZ016) | 100.00 | 100.00 |
| TC-B-614 | *A. phagocytophilum* | OL690562.1 | 99.00 | 100.00 |
| YB-B-36 | *A. platys* | [MF289478.1](https://www.ncbi.nlm.nih.gov/nucleotide/MF289478.1?report=genbank&log$=nucltop&blast_rank=13&RID=WTR4FZEG013) | 100.00 | 99.29 |
| TC-B-567 | *A. platys* | [MF289478.1](https://www.ncbi.nlm.nih.gov/nucleotide/MF289478.1?report=genbank&log$=nucltop&blast_rank=13&RID=WTR4FZEG013) | 100.00 | 99.83 |
| TC-B-482 | *A. platys* | [MF289478.1](https://www.ncbi.nlm.nih.gov/nucleotide/MF289478.1?report=genbank&log$=nucltop&blast_rank=13&RID=WTR4FZEG013) | 100.00 | 99.16 |
| TC-B-721 | *A.platys* | [OK560282.1](https://www.ncbi.nlm.nih.gov/nucleotide/OK560282.1?report=genbank&log$=nucltop&blast_rank=16&RID=WVC4EF7B016) | 99.00 | 98.31 |
| TC-B-729 | *A. platys* | [OK560282.1](https://www.ncbi.nlm.nih.gov/nucleotide/OK560282.1?report=genbank&log$=nucltop&blast_rank=1&RID=WVWCR62S016) | 99.00 | 98.48 |
| TC-B-737 | *A. platys* | [OK560281.1](https://www.ncbi.nlm.nih.gov/nucleotide/OK560281.1?report=genbank&log$=nucltop&blast_rank=1&RID=WVVRH9N3016) | 99.00 | 98.65 |
| TC-B-598 | *A. platys* | [MK506833.1](https://www.ncbi.nlm.nih.gov/nucleotide/MK506833.1?report=genbank&log$=nucltop&blast_rank=4&RID=WVVSGPRG016) | 100.00 | 98.49 |
| TC-B-727 | *A. marginale* | [MK804764.1](https://www.ncbi.nlm.nih.gov/nucleotide/MK804764.1?report=genbank&log$=nucltop&blast_rank=1&RID=WVWR4UTX016) | 100.00 | 100.00 |
| YJ-B-5 | *A. marginale* | [MK804764.1](https://www.ncbi.nlm.nih.gov/nucleotide/MK804764.1?report=genbank&log$=nucltop&blast_rank=1&RID=WVWR4UTX016) | 100.00 | 98.51 |
| DQ-B-19 | *A. marginale* | [MK804764.1](https://www.ncbi.nlm.nih.gov/nucleotide/MK804764.1?report=genbank&log$=nucltop&blast_rank=1&RID=WVWEDHFN016) | 100.00 | 99.60 |
| XX-B-37 | *A. marginale* | [MK804764.1](https://www.ncbi.nlm.nih.gov/nucleotide/MK804764.1?report=genbank&log$=nucltop&blast_rank=1&RID=WVWEDHFN016) | 100.00 | 99.80 |
| DY-B-5 | *A. marginale* | [MK804764.1](https://www.ncbi.nlm.nih.gov/nucleotide/MK804764.1?report=genbank&log$=nucltop&blast_rank=1&RID=WVWEDHFN016) | 100.00 | 99.80 |
| TC-B-479 | *A. capra* | MG869594.1 | 100.00 | 100.00 |
| TC-B-240 | *A. bovis* | [MH255940.1](https://www.ncbi.nlm.nih.gov/nucleotide/MH255940.1?report=genbank&log$=nucltop&blast_rank=3&RID=WVWX881E01N) | 100.00 | 98.56 |
| DQ-B-3 | *A. bovis* | [MH255940.1](https://www.ncbi.nlm.nih.gov/nucleotide/MH255940.1?report=genbank&log$=nucltop&blast_rank=3&RID=WVWX881E01N) | 100.00 | 99.46 |
| TC-B-243 | *A. bovis* | [MH255940.1](https://www.ncbi.nlm.nih.gov/nucleotide/MH255940.1?report=genbank&log$=nucltop&blast_rank=3&RID=WVWX881E01N) | 100.00 | 100.00 |
| GM-B-38 | *A. ovis* | MG869525.1 | 100.00 | 100.00 |
| HP-B-8 | *A. ovis* | MG869525.1 | 100.00 | 100.00 |
| JC-B-47 | *A. ovis* | MG869525.1 | 100.00 | 100.00 |
| LP-B-10 | *A. ovis* | MG869525.1 | 100.00 | 100.00 |
| TC-B-182 | *A. ovis* | MG869525.1 | 100.00 | 100.00 |
| Wsh-B-09 | *A. ovis* | MG869525.1 | 100.00 | 100.00 |
| YunL-B-10 | *A. ovis* | [KJ410244.1](https://www.ncbi.nlm.nih.gov/nucleotide/KJ410244.1?report=genbank&log$=nucltop&blast_rank=1&RID=WVX92SW701N) | 100.00 | 99.83 |
| YunL-B-122 | *Ehrlichia cains* | [MK507008.1](https://www.ncbi.nlm.nih.gov/nucleotide/MK507008.1?report=genbank&log$=nucltop&blast_rank=4&RID=WVXF3JVU01N) | 100.00 | 99.50 |
| YunL-B-120 | *Ehrlichia cains* | [MK507008.1](https://www.ncbi.nlm.nih.gov/nucleotide/MK507008.1?report=genbank&log$=nucltop&blast_rank=4&RID=WVXF3JVU01N) | 100.00 | 99.43 |
| TC-B-635 | *Ehrlichia cains* | [MK507008.1](https://www.ncbi.nlm.nih.gov/nucleotide/MK507008.1?report=genbank&log$=nucltop&blast_rank=4&RID=WVXF3JVU01N) | 100.00 | 99.33 |
| LP-B-2 | *Ehrlichia cains* | [MK507008.1](https://www.ncbi.nlm.nih.gov/nucleotide/MK507008.1?report=genbank&log$=nucltop&blast_rank=4&RID=WVXF3JVU01N) | 100.00 | 99.29 |
| DY-B-13 | *Ehrlichia cains* | [MK507008.1](https://www.ncbi.nlm.nih.gov/nucleotide/MK507008.1?report=genbank&log$=nucltop&blast_rank=4&RID=WVXF3JVU01N) | 100.00 | 98.86 |
| MLa-B-22 | *Wolbachia pipientis* | [CP037426.1](https://www.ncbi.nlm.nih.gov/nucleotide/CP037426.1?report=genbank&log$=nucltop&blast_rank=1&RID=WVXHG91501N) | 100.00 | 99.83 |
| TC-T-49 | *A. phagocytophilum* | [OL690565.1](https://www.ncbi.nlm.nih.gov/nucleotide/OL690565.1?report=genbank&log$=nuclalign&blast_rank=1&RID=WVXJ72B7013) | 100.00 | 99.47 |
| TC-T-157 | *A. platys* | [MK736884.1](https://www.ncbi.nlm.nih.gov/nucleotide/MK736884.1?report=genbank&log$=nucltop&blast_rank=1&RID=WVYP5W12013) | 99.00 | 99.23 |
| LS-T-35 | *A. platys* | [MF289478.1](https://www.ncbi.nlm.nih.gov/nucleotide/MF289478.1?report=genbank&log$=nucltop&blast_rank=15&RID=WVYPMGU2013) | 100.00 | 99.30 |
| HQ-T-1 | *Anaplasma* sp. strain HQT1 | [MK814441.1](https://www.ncbi.nlm.nih.gov/nucleotide/MK814441.1?report=genbank&log$=nucltop&blast_rank=1&RID=WVYR9CRA013) | 100.00 | 98.49 |
| JC-T-222 | *Anaplasma* sp. strain JCT222 | [KY924886.1](https://www.ncbi.nlm.nih.gov/nucleotide/KY924886.1?report=genbank&log$=nucltop&blast_rank=1&RID=WVYRRDBK013) | 100.00 | 100.00 |
| DQ-T-52 | *A. bovis* | [MH255939.1](https://www.ncbi.nlm.nih.gov/nucleotide/MH255939.1?report=genbank&log$=nuclalign&blast_rank=1&RID=WVYS8YGY013) | 100.00 | 99.73 |
| TC-T-400 | *Anaplasma* sp. Strain TCT400 | [OL690561.1](https://www.ncbi.nlm.nih.gov/nucleotide/OL690561.1?report=genbank&log$=nucltop&blast_rank=1&RID=WVYST202013) | 99.00 | 97.80 |
| JH-T-19 | *A. marginale* | [MK804764.1](https://www.ncbi.nlm.nih.gov/nucleotide/MK804764.1?report=genbank&log$=nuclalign&blast_rank=1&RID=WVZ9F7F701N) | 100.00 | 100.00 |
| TC-T-398 | *A. ovis* | [OR214930.1](https://www.ncbi.nlm.nih.gov/nucleotide/OR214930.1?report=genbank&log$=nucltop&blast_rank=1&RID=WVZ9W5MH01N) | 100.00 | 99.83 |
| YunL-T-18 | *A. ovis* | [KJ410245.1](https://www.ncbi.nlm.nih.gov/nucleotide/KJ410245.1?report=genbank&log$=nucltop&blast_rank=1&RID=WVZAEN2J013) | 99.00 | 100.00 |
| YuL-T-53 | *A. ovis* | [MG869525.1](https://www.ncbi.nlm.nih.gov/nucleotide/MG869525.1?report=genbank&log$=nucltop&blast_rank=1&RID=WVZAUG2W013) | 100.00 | 100.00 |
| JC-T-88 | *A. ovis* | [MG869525.1](https://www.ncbi.nlm.nih.gov/nucleotide/MG869525.1?report=genbank&log$=nucltop&blast_rank=1&RID=WVZAUG2W013) | 100.00 | 100.00 |
| HQ-T-7 | *A. ovis* | [MG869525.1](https://www.ncbi.nlm.nih.gov/nucleotide/MG869525.1?report=genbank&log$=nucltop&blast_rank=1&RID=WVZAUG2W013) | 100.00 | 100.00 |
| DQ-T-56 | *A. ovis* | [MG869525.1](https://www.ncbi.nlm.nih.gov/nucleotide/MG869525.1?report=genbank&log$=nucltop&blast_rank=1&RID=WVZAUG2W013) | 100.00 | 100.00 |
| JC-T-246 | *Ehrlichia cains* | [MK507008.1](https://www.ncbi.nlm.nih.gov/nucleotide/MK507008.1?report=genbank&log$=nucltop&blast_rank=8&RID=WVZCFPAH013) | 100.00 | 99.00 |
| TC-T-403 | *Ehrlichia cains* | [MK507008.1](https://www.ncbi.nlm.nih.gov/nucleotide/MK507008.1?report=genbank&log$=nucltop&blast_rank=8&RID=WVZCFPAH013) | 100.00 | 98.67 |
| TC-T-393 | *Ehrlichia cains* | [MK507008.1](https://www.ncbi.nlm.nih.gov/nucleotide/MK507008.1?report=genbank&log$=nucltop&blast_rank=8&RID=WVZCFPAH013) | 100.00 | 98.84 |
| LS-T-33 | *Ehrlichia cains* | [MK507008.1](https://www.ncbi.nlm.nih.gov/nucleotide/MK507008.1?report=genbank&log$=nucltop&blast_rank=8&RID=WVZCFPAH013) | 100.00 | 99.00 |
| JC-T-251 | *Ehrlichia cains* | [MK507008.1](https://www.ncbi.nlm.nih.gov/nucleotide/MK507008.1?report=genbank&log$=nucltop&blast_rank=8&RID=WVZCFPAH013) | 100.00 | 99.34 |
| YunL-T-26 | *Candidatus* Neoehrlichia mikurensis | [AB196304.1](https://www.ncbi.nlm.nih.gov/nucleotide/AB196304.1?report=genbank&log$=nucltop&blast_rank=1&RID=WW0A1GJM013) | 100.00 | 99.64 |
| YunL-T-13 | *Candidatus* Neoehrlichia mikurensis | [AB196304.1](https://www.ncbi.nlm.nih.gov/nucleotide/AB196304.1?report=genbank&log$=nucltop&blast_rank=1&RID=WW0A1GJM013) | 100.00 | 99.46 |
| HQ-T-2 | *R. hoogstraalii* | [NR_104877.1](https://www.ncbi.nlm.nih.gov/nucleotide/NR_104877.1?report=genbank&log$=nucltop&blast_rank=1&RID=WW0B515V016) | 100.00 | 99.83 |
| EY-T-6 | *R. hoogstraalii* | [NR_104877.1](https://www.ncbi.nlm.nih.gov/nucleotide/NR_104877.1?report=genbank&log$=nucltop&blast_rank=1&RID=WW0B515V016) | 100.00 | 99.83 |
| YunL-T-70 | *R. hoogstraalii* | [NR_104877.1](https://www.ncbi.nlm.nih.gov/nucleotide/NR_104877.1?report=genbank&log$=nucltop&blast_rank=1&RID=WW0B515V016) | 100.00 | 99.83 |
| JC-T-62 | *Rickettsia massiliae* | [CP003319.1](https://www.ncbi.nlm.nih.gov/nucleotide/KF318168.1?report=genbank&log$=nucltop&blast_rank=1&RID=WW0RDNZM016) | 100.00 | 100.00 |
| EY-T-16 | *Rickettsia massiliae* | [CP003319.1](https://www.ncbi.nlm.nih.gov/nucleotide/KF318168.1?report=genbank&log$=nucltop&blast_rank=1&RID=WW0RDNZM016) | 100.00 | 100.00 |
| JH-T-15 | *Rickettsia massiliae* | [CP003319.1](https://www.ncbi.nlm.nih.gov/nucleotide/KF318168.1?report=genbank&log$=nucltop&blast_rank=1&RID=WW0RDNZM016) | 100.00 | 100.00 |
| YunL-T-67 | *Candidatus R. longicornii* | [MT747412.1](https://www.ncbi.nlm.nih.gov/nucleotide/MT747412.1?report=genbank&log$=nucltop&blast_rank=21&RID=WW0SHM60013) | 100.00 | 99.83 |
| YunL-T-49 | *Candidatus R. longicornii* | [MT747412.1](https://www.ncbi.nlm.nih.gov/nucleotide/MT747412.1?report=genbank&log$=nucltop&blast_rank=21&RID=WW0SHM60013) | 100.00 | 99.83 |
| YunL-T-30 | *Candidatus R. longicornii* | [MT747412.1](https://www.ncbi.nlm.nih.gov/nucleotide/MT747412.1?report=genbank&log$=nucltop&blast_rank=21&RID=WW0SHM60013) | 100.00 | 100.00 |
| WX-T-85 | *Candidatus R. longicornii* | [MG906672.1](https://www.ncbi.nlm.nih.gov/nucleotide/MG906672.1?report=genbank&log$=nucltop&blast_rank=1&RID=WW0TRB1E013) | 100.00 | 100.00 |
| TC-T-155 | *Candidatus R. longicornii* | [MG906672.1](https://www.ncbi.nlm.nih.gov/nucleotide/MG906672.1?report=genbank&log$=nucltop&blast_rank=1&RID=WW0TRB1E013) | 100.00 | 99.30 |
| LS-T-38 | *Candidatus R. longicornii* | [MG906672.1](https://www.ncbi.nlm.nih.gov/nucleotide/MG906672.1?report=genbank&log$=nucltop&blast_rank=1&RID=WW0TRB1E013) | 100.00 | 99.30 |
| JC-T-135 | *Candidatus R. longicornii* | [MG906672.1](https://www.ncbi.nlm.nih.gov/nucleotide/MG906672.1?report=genbank&log$=nucltop&blast_rank=1&RID=WW0TRB1E013) | 100.00 | 100.00 |
| GM-T-167 | *Candidatus R. longicornii* | MT747412.1 | 100.00 | 100.00 |
| EY-T-3 | *Candidatus R. longicornii* | [MG906672.1](https://www.ncbi.nlm.nih.gov/nucleotide/MG906672.1?report=genbank&log$=nucltop&blast_rank=1&RID=WW0TRB1E013) | 100.00 | 100.00 |
| DQ-T-40 | *Candidatus R. longicornii* | MT747412.1 | 100.00 | 99.82 |


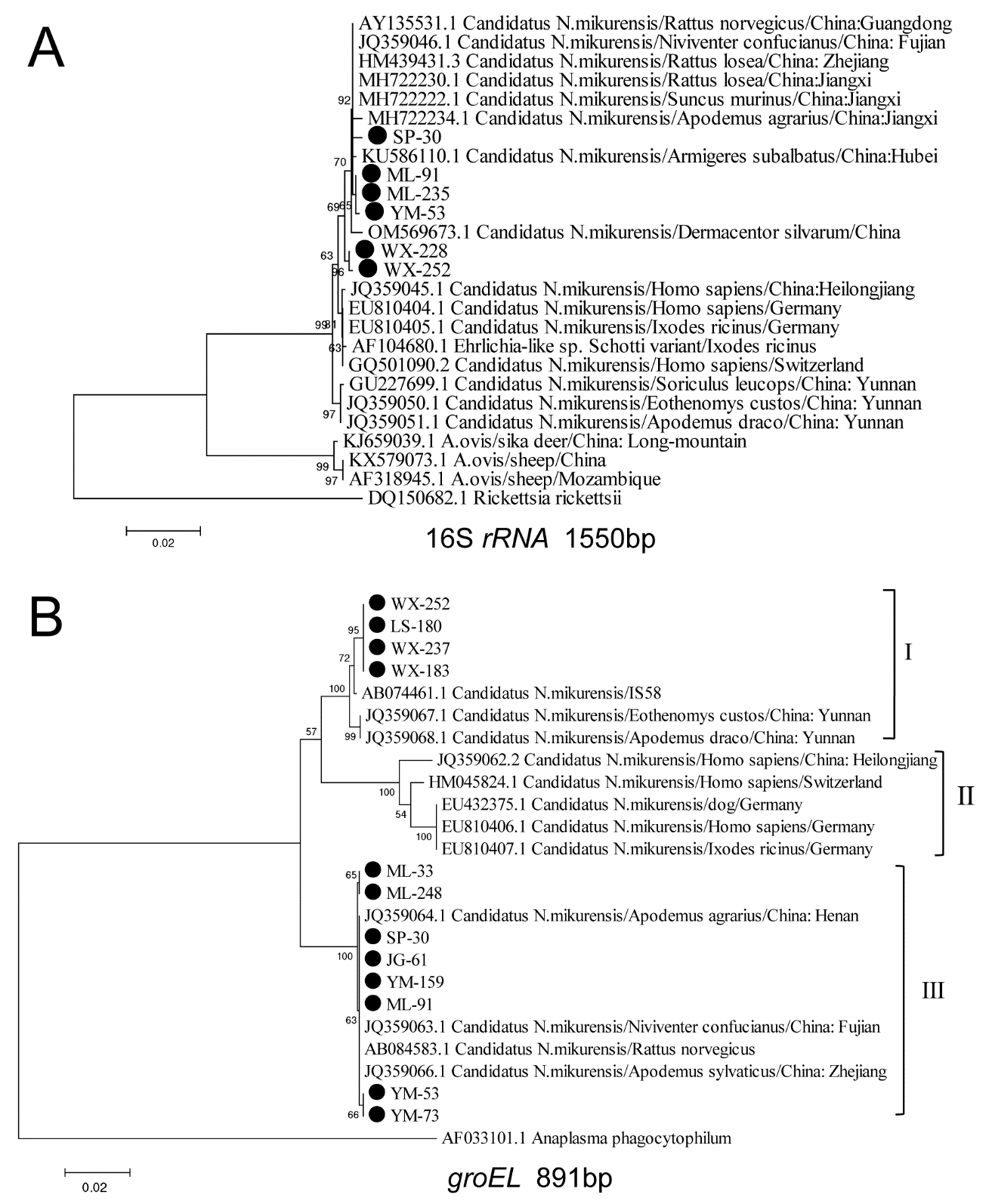
Figure S1. Phylogenetic trees constructed by the MEGA v.6.0 software based on the neighbor-joining method of multigene detected in small mammals infected by *Candidatus* Neoehrlichia mikurensis. A: 16S rRNA gene (1550 bp); B: *groEL* gene (891 bp).


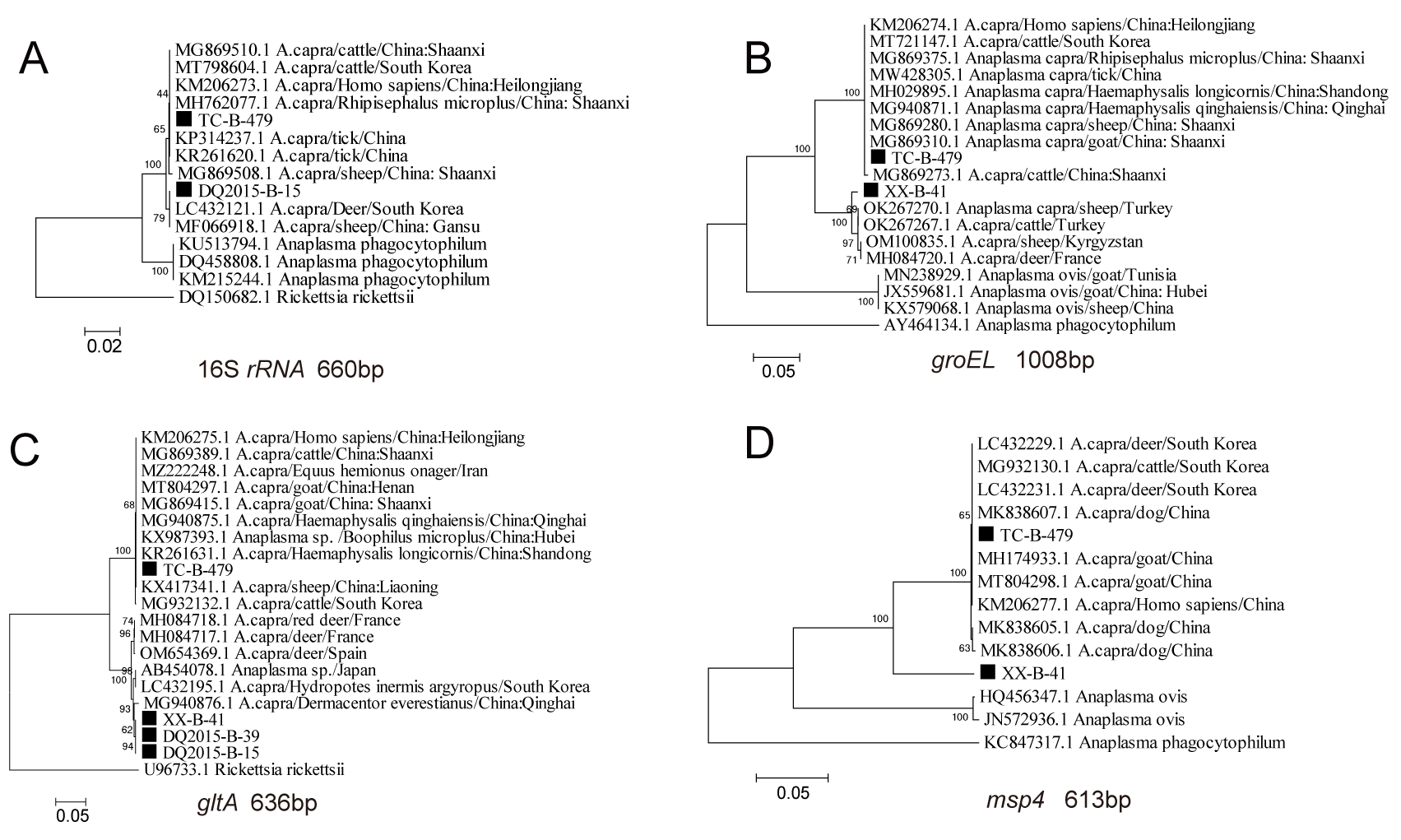
Figure S2. Phylogenetic trees constructed by the MEGA v.6.0 software based on the neighbor-joining method of multigene detected in livestock infected by *Anaplasma capra*. A: 16S rRNA gene (660 bp); B: *groEL* gene (1008 bp); C: *gltA* gene (636 bp); D: *msp4* gene (613 bp)/


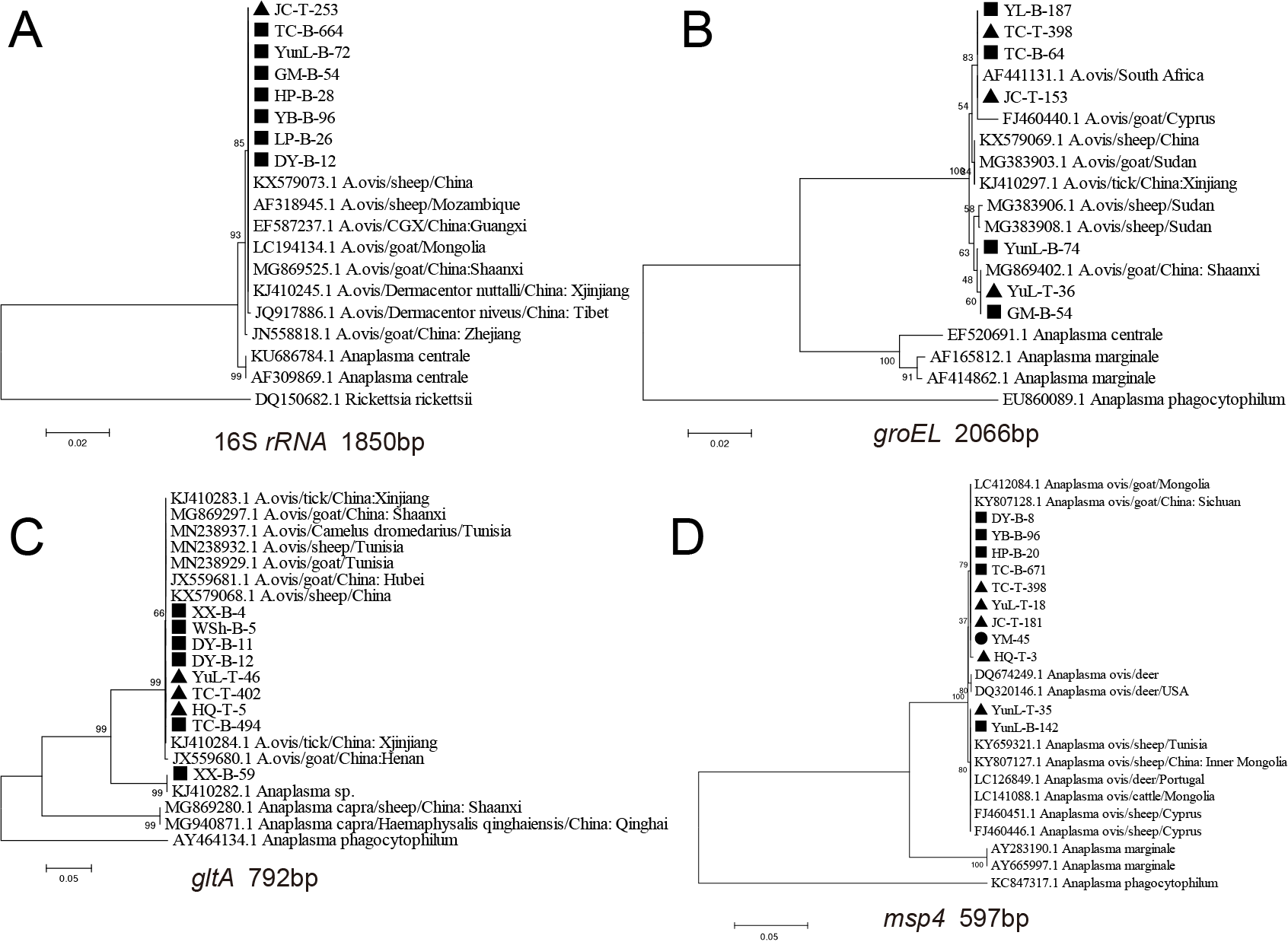


Figure S3. Phylogenetic trees constructed by the MEGA v.6.0 software based on the Neighbor-Joining method of multigene detected in different samples infected *Anaplasma ovis*. Triangles, circles and rectangles represent livestock, small mammals, and ticks in this study respectively. A: 16S rRNA gene (1850 bp); B: *groEL* gene (2066 bp); C: *gltA* gene (792 bp); D: *msp4* gene (597 bp).


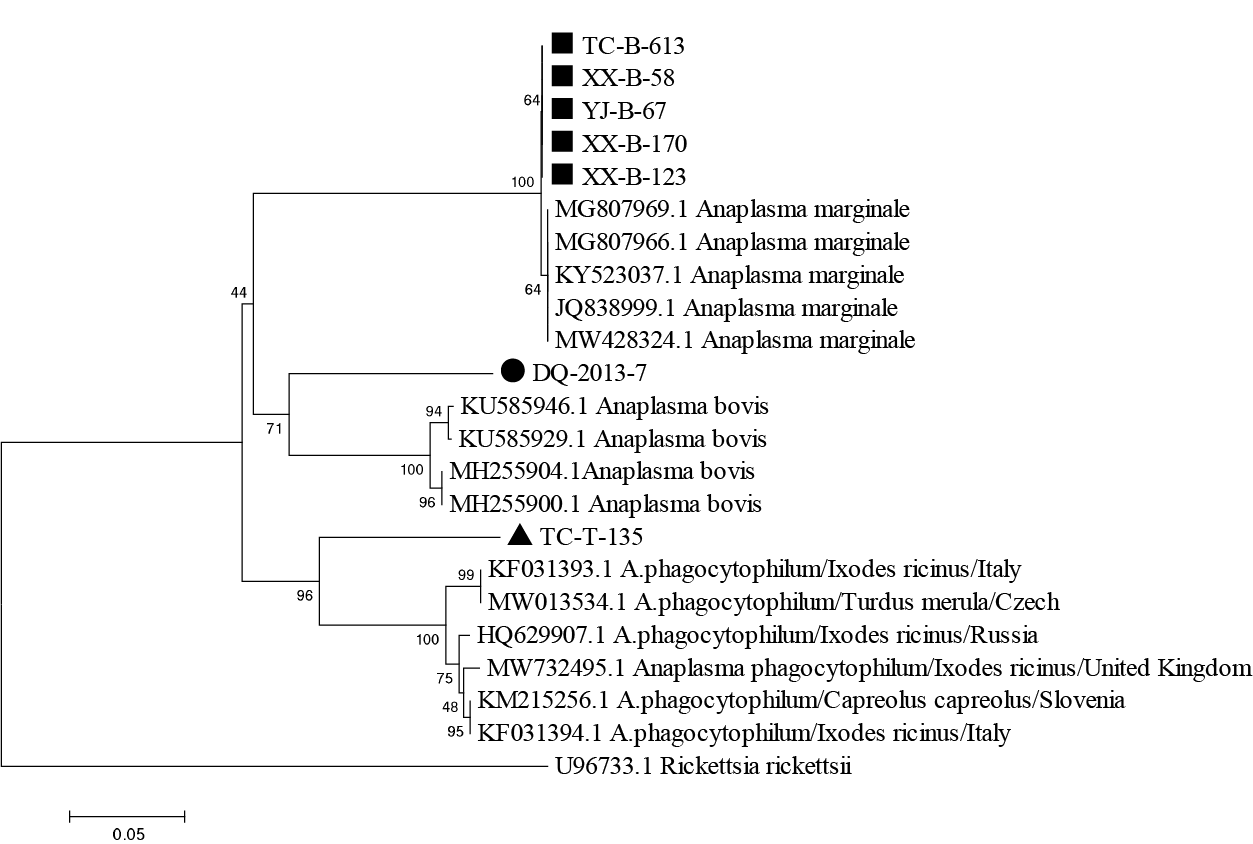


Figure S4. Phylogenetic trees constructed by the MEGA v.6.0 software based on the neighbor-joining method of *groEL* gene (372 bp) detected in different samples. Triangles, circles and rectangles represent livestock, small mammals, and ticks in this study respectively.


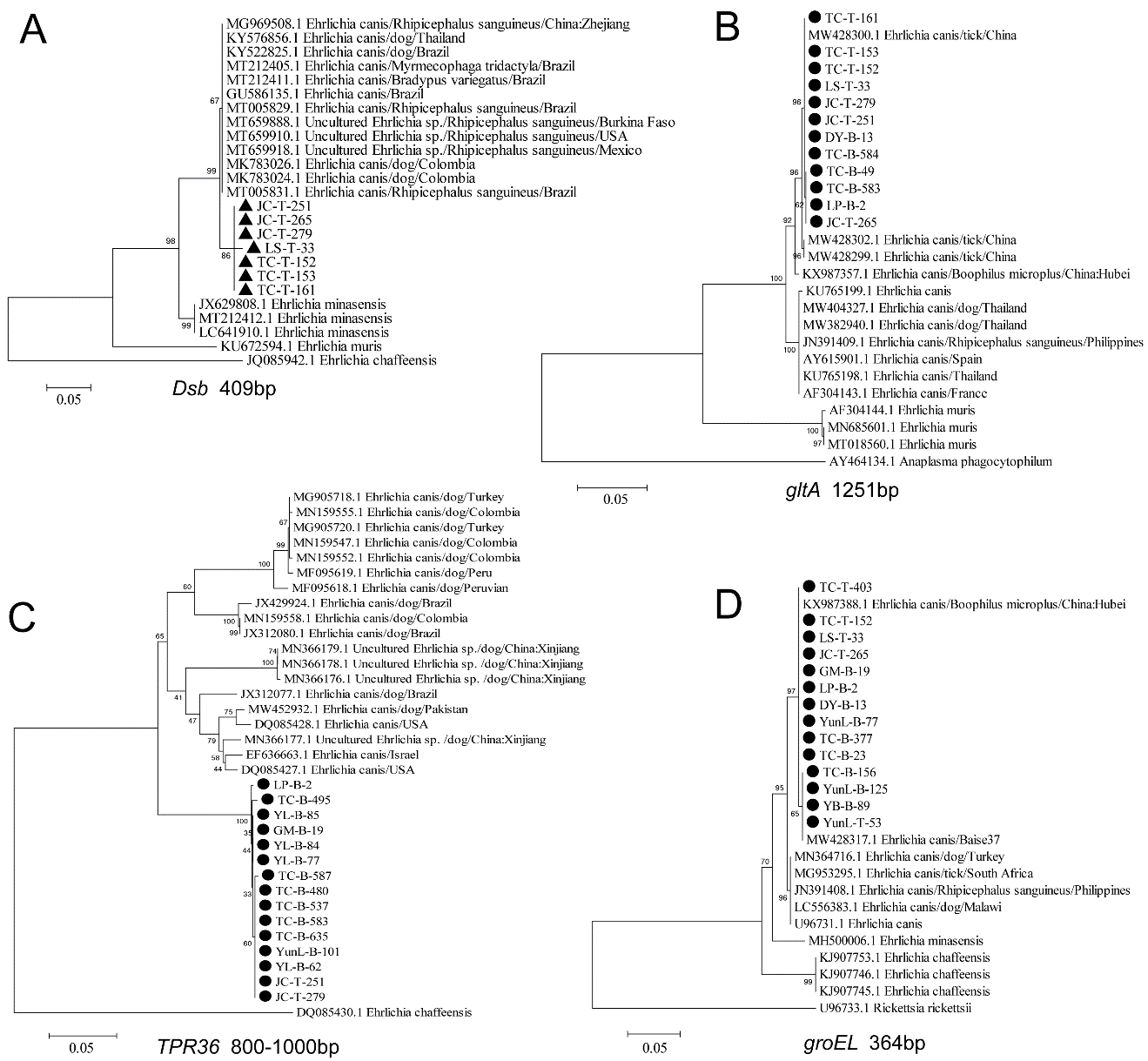
Figure S5. Phylogenetic trees constructed by the MEGA v.6.0 software based on the neighbor-joining method of multigene detected in different samples infected *Ehrlichia canis*. Circles and rectangles represent small mammals and ticks in this study respectively. A: *Dsb* gene (409 bp); B: *gltA* gene (125 bp); C: *TRP36* gene (800–1000 bp); D: *groEL* gene (364 bp).
